# Physics-Constrained Transfer-Matrix Optimization of Diffused Regions in Bifacial *p*^+^–*n*–*n*^+^ Crystalline-Silicon Solar Cells

**DOI:** 10.3390/nano16181187

**Published:** 2026-09-20

**Authors:** Pablo Ferrada, Carlos Portillo

**Affiliations:** 1 Departamento de Ingeniería, Universidad Loyola Andalucía, Av. de las Universidades, 2, 41704 Dos Hermanas, Spain; 2 Instituto de Investigación en Energía, Tecnología y Sostenibilidad (LETS), Universidad Loyola Andalucía, Av. de las Universidades, 2, 41704 Dos Hermanas, Spain; 3 Centro de Desarrollo Energético Antofagasta (CDEA), Universidad de Antofagasta, Av. Angamos 601, Antofagasta 1270300, Chile; carlos.portillo@uantof.cl

**Keywords:** crystalline-silicon solar cells, transfer-matrix method, drift–diffusion model, minority-carrier transport, complementary error-function doping profiles, physics-constrained optimization, sheet resistance, bifacial n-PERT cells

## Abstract

Physics-based models are useful for the analysis and optimization of crystalline-silicon solar cells when many device designs must be evaluated. This paper presents a compact transfer-matrix framework for one-dimensional carrier transport and physics-constrained optimization in bifacial $p^{+}$–*n*–$n^{+}$ crystalline-silicon solar cells under front-side illumination. The model includes a distributed optical generation profile, explicit emitter, base, and rear-field regions, surface recombination, doping-dependent mobility, Auger recombination, band-gap narrowing, sheet and contact resistances, and external series and shunt losses. The front and rear dopant distributions are described by complementary-error-function profiles, so their junction depths follow from the diffusion parameters and base concentration instead of being independent optimization variables. The solver is verified in limiting transport conditions and with tabulated generation data. The complete model is then calibrated against the reported photovoltaic figures of merit of an experimental bifacial n-type passivated-emitter and rear-totally-diffused (n-PERT) solar cell, which serves as the reference device and is subsequently applied to global optimization. The calibration reproduces a short-circuit current density of $J_{sc}=39.20\;\mathrm{mA}/{\mathrm{cm}}^{2}$, an open-circuit voltage of $V_{oc}=653.1\;\mathrm{mV}$, a fill factor of 0.783, and an efficiency of 20.05%. For the 180 µm reference geometry, the optimized design reaches an efficiency of 20.63%. Independent optimizations for wafer thicknesses of 160, 180, and 200 µm produce a consistent family of solutions with efficiency gains of approximately 0.56–0.60 percentage points. These gains result from the higher open-circuit voltage and fill factor despite a moderate reduction in short-circuit current density, while the optimized total series resistance remains nearly constant. The framework provides a physically interpretable method for combining carrier-transport modeling, experimental calibration, inverse design, and repeated global optimization.

## 1. Introduction

Crystalline-silicon photovoltaics is the dominant technology for large-scale solar-energy conversion and represents most of the current photovoltaic market [1]. Further improvements in solar-cell performance require the simultaneous control of optical absorption, carrier transport, bulk and surface recombination, contact behavior, and resistive losses [2,3]. These mechanisms are strongly coupled. Changes in wafer thickness, base doping, or front and rear diffusion profiles may improve carrier collection or reduce electrical resistance, but they may also increase recombination or contact-related losses [4]. Therefore, the design of high-performance crystalline-silicon solar cells requires the combined analysis of several physical and geometric parameters.

Beyond crystalline silicon, photovoltaic research includes several other material systems, such as thin-film semiconductors, III–V compounds, halide perovskites, and oxide-based functional materials. Binary oxides such as TiO_2_, ZnO, Al_2_O_3_, SiO_2_, CeO_2_, Fe_2_O_3_, and WO_3_ can play different roles in silicon, perovskite, dye-sensitized, and thin-film solar cells [5]. Halide perovskites are another important class of photovoltaic materials, although their long-term stability remains a major research challenge, especially under demanding environmental conditions [6]. Within this broader photovoltaic materials landscape, crystalline silicon provides a well-established platform for examining the coupled effects of carrier transport, recombination, and electrical resistance in doped regions.

Physics-based numerical models allow these interactions to be studied before fabrication and can reduce the number of experimental iterations required during device development [7]. Drift–diffusion models connect material properties and device geometry with internal carrier distributions and terminal current–voltage characteristics [8]. They can therefore be used for forward simulation, experimental calibration, sensitivity analysis, and numerical optimization. However, calibration and global optimization usually require many repeated forward simulations. This creates a practical need for models that retain the relevant semiconductor physics while remaining sufficiently compact for systematic parameter exploration.

Detailed commercial and research-oriented device simulators provide well-established tools for crystalline-silicon solar-cell analysis. TCAD and finite-element formulations can include multidimensional electrostatics, detailed recombination models, optical solvers, contact descriptions, and realistic device geometries [9,10,11]. Mature solar-cell simulation environments such as PC1D and Quokka also provide established approaches for one-dimensional and reduced-order device modeling [12,13]. Reduced-order approaches have also been developed for specific parts of the device model, including simplified optical descriptions for silicon solar cells [14]. These tools and formulations have been widely used to study how material, geometric, and electrical parameters affect solar-cell performance.

Recent studies further illustrate the role of numerical simulation in photovoltaic design optimization. Quokka-based analyses have been used to investigate wafer properties, carrier lifetime, resistivity, and geometrical design variables in high-efficiency crystalline-silicon architectures [15]. PC1D and AFORS-HET have also been applied to the systematic optimization of silicon-cell structures and material parameters [16]. In addition, recent drift–diffusion fitting studies have shown that calibration can become difficult when many free parameters are correlated, highlighting the importance of using physically constrained parameter spaces [17]. These studies show the value of numerical models for repeated parameter exploration and motivate formulations in which the main physical relationships remain explicit during optimization.

The drift–diffusion framework for semiconductor devices is itself well established [18]. In quasi-neutral regions and under low-injection conditions, minority-carrier transport can be described by diffusion–reaction equations. Analytical solutions are available for uniform layers, constant material properties, and simple generation profiles [19]. For layered one-dimensional systems, matrix-based approaches provide a compact alternative in which each homogeneous region is solved separately and the complete structure is assembled through interface and boundary conditions. Related matrix formulations have been applied directly to minority-carrier transport in multilayer semiconductor structures [20]. Transfer- and scattering-matrix approaches have also been used in the photovoltaic modeling of multilayer and multijunction solar cells together with analytical solutions of the semiconductor continuity equations [21]. More generally, the same mathematical idea of propagating a state variable and its flux through consecutive layers also appears in diffusion–reaction problems outside semiconductor physics [22].

For photovoltaic design, however, a layer-wise transport model must also remain connected to the other physical mechanisms that determine the electrical response of the device. This requires spatially varying optical generation to be included in the carrier-transport problem, consistent treatment of the emitter, base, and rear-field regions, and appropriate boundary conditions for surface recombination. In addition, mobility, carrier lifetime, recombination, and electrical resistance depend on doping [23]. These relationships become particularly important during optimization because dopant concentration, junction depth, carrier transport, recombination, and resistive losses cannot be varied independently without affecting the physical consistency of the device.

To investigate these interactions, we consider a bifacial $p^{+}$–*n*–$n^{+}$ crystalline-silicon solar cell based on the experimental n-PERT device reported in Ref. [24]. The structure contains a front $p^{+}$ emitter, an *n*-type base, and a rear $n^{+}$ field. In the reference device, the front and rear junction depths lie in the submicrometer range, so device performance is strongly influenced by thin doped regions close to both surfaces. These thin doped regions are functional parts of the device rather than purely geometric features: their dopant profiles affect carrier transport, bulk and surface recombination, sheet resistance, and contact behavior [25,26,27]. For constant-source thermal diffusion in crystalline silicon, Fick’s second law leads to a complementary-error-function dopant profile with the junction depth defined by the position at which the diffused dopant concentration reaches the background doping level [18,28]. The rear diffusion profile and metallization geometry also affect lateral current-collection losses [29]. Their material properties and dopant distributions therefore play a direct role in the electrical response of the complete device.

The same n-PERT device was previously analyzed in Ref. [24] using the Semiconductor Module of COMSOL Multiphysics together with a genetic algorithm. In that study, the one-dimensional drift–diffusion problem was solved numerically, and the wafer, emitter, and rear-field thicknesses and their corresponding doping concentrations were treated as independent design variables. Recombination losses were represented mainly through volumetric mechanisms within the semiconductor domains, while explicit front and rear surface-recombination velocities were not included as boundary parameters.

The main methodological differences with respect to Ref. [24] can be summarized as follows: (i) carrier transport is described through explicit transfer-matrix blocks for the $p^{+}$ emitter, *n*-type base, and $n^{+}$ rear field instead of solving the full one-dimensional drift–diffusion problem with a finite-element semiconductor solver; (ii) front and rear surface recombination is introduced explicitly through Robin boundary conditions, separating surface losses from volumetric recombination; (iii) the front and rear dopant distributions are described by complementary-error-function profiles, so the emitter and rear-field junction depths are derived from the diffusion profiles and base concentration instead of being independent optimization variables; (iv) for each prescribed wafer thickness, the independent design variables are the front surface doping, base doping, and rear surface doping, while the corresponding junction depths are calculated from the physical diffusion constraints; and (v) the global search is followed by a local SQP refinement, while Ref. [24] used a genetic-algorithm search.

These changes reduce the number of independent geometrical variables and keep doping, junction depth, carrier transport, surface and bulk recombination, and resistive losses physically linked during optimization. The use of the same experimental n-PERT cell provides a common reference for comparing the two modeling approaches.

The contribution of this paper is the integration of these elements into a reduced-order transfer-matrix framework for the experimentally anchored and physics-constrained inverse design of layered $p^{+}$–*n*–$n^{+}$ crystalline-silicon solar cells. The transfer-matrix method itself is well established; here, it is used as the basis of a compact formulation that connects layer-wise minority-carrier transport with distributed generation, surface recombination, diffusion profiles, doping-dependent losses, calibration, and global optimization. Once the regional matrices and source vectors are constructed for a candidate design, the voltage sweep is reduced to small matrix operations and algebraic device closure.

The solver is first verified in limiting transport cases and with tabulated optical generation. The complete model is then calibrated against the reported photovoltaic figures of merit of the experimental n-PERT reference cell and applied to global optimization for prescribed wafer thicknesses of 160, 180, and 200 µm. These independent cases are used to study the consistency of the optimized diffusion profiles and loss balances under controlled changes in wafer thickness rather than to determine an industrially optimal wafer thickness.

## 2. Materials and Methods

### 2.1. Transfer-Matrix Carrier-Transport Model

The one-dimensional device considered in this paper contains three quasi-neutral transport regions: the front $p^{+}$ emitter (*E*), the *n*-type base (*B*), and the rear $n^{+}$ back-surface-field region (*S*). The generic index r∈{E,B,S} refers to any one of these regions.

The global depth coordinate *x* is measured from the front surface, and the complete device occupies $0\leq x\leq d_{0}$, where $d_{0}$ is the prescribed wafer thickness. For region *r*, the symbol $a_{r}$ denotes its starting position and $\ell_{r}$ its physical thickness. In the present device, the emitter starts at $a_{E}=0$, the base at $a_{B}=W_{E}$, and the BSF at $a_{S}=W_{E}+W_{B}$. Their corresponding thicknesses are $\ell_{E}=W_{E}$, $\ell_{B}=W_{B}$, and $\ell_{S}=W_{\mathrm{BSF}}$.

A local coordinate $x^{\prime }$ is measured from the start of each region, so that $0\leq x^{\prime }\leq \ell_{r}$ and the corresponding global depth is $x=a_{r}+x^{\prime }$.

In each region, the stationary minority-carrier diffusion–reaction Equation is [30]

(1) $$D_{r}\frac{d^{2}\Delta c_{r}}{d{x^{\prime }}^{2}}-\frac{\Delta c_{r}}{\tau_{r}}+g_{r}\left(x^{\prime }\right)=0,\;0<x^{\prime }<\ell_{r},$$

where $\Delta c_{r}\left(x^{\prime }\right)$ is the excess minority-carrier concentration, $D_{r}$ is the minority-carrier diffusion coefficient, $\tau_{r}$ is the effective minority-carrier lifetime, and $g_{r}\left(x^{\prime }\right)$ is the local electron–hole-pair generation rate. For a global generation profile g(x), the local profile is $g_{r}\left(x^{\prime }\right)=g\left(a_{r}+x^{\prime }\right)$. The equilibrium minority-carrier concentration in region *r* is denoted by $c_{0,r}$. The normalized excess concentration $u_{r}$ and the diffusion length $L_{r}$ are defined as

(2) $$u_{r}\left(x^{\prime }\right)=\frac{\Delta c_{r}\left(x^{\prime }\right)}{c_{0,r}},\;L_{r}=\sqrt{D_{r}\tau_{r}}.$$

The scaled diffusion flux $G_{r}$ and the two-component carrier-state vector $s_{r}$ are

(3) $$G_{r}\left(x^{\prime }\right)=D_{r}c_{0,r}\frac{du_{r}}{dx^{\prime }},\;s_{r}\left(x^{\prime }\right)=\left(\begin{array}{c} u_{r}\left(x^{\prime }\right)\\ G_{r}\left(x^{\prime }\right) \end{array}\right).$$

The quantities $u_{r}$ and $G_{r}$ are scalar fields. Bold lowercase symbols, such as $s_{r}$ and $b_{r}$, denote column vectors, while uppercase symbols, such as $M_{r}$, denote matrices. The first component of $s_{r}$ is the normalized excess minority-carrier concentration. The second component is proportional to the diffusion flux. This state representation allows concentration and carrier flow to be propagated together through each region [20,22].

The symbol Δx denotes the physical distance covered in one propagation step inside region *r*. If propagation starts at the local position $x^{\prime }=a$ and ends at $x^{\prime }=b$, then Δx=b−a. For propagation across the complete region, a=0, $b=\ell_{r}$, and therefore $\Delta x=\ell_{r}$. The transfer matrix for a propagation distance Δx is

(4) $$M_{r}\left(\Delta x\right)=\left(\begin{array}{cc} \cosh\;\left(\frac{\Delta x}{L_{r}}\right)\; & \frac{L_{r}}{D_{r}c_{0,r}}\sinh\;\left(\frac{\Delta x}{L_{r}}\right)\\ \frac{D_{r}c_{0,r}}{L_{r}}\sinh\;\left(\frac{\Delta x}{L_{r}}\right)\; & \cosh\;\left(\frac{\Delta x}{L_{r}}\right) \end{array}\right).$$

Thus, the argument of $M_{r}$ is a physical distance, while the hyperbolic functions depend on the dimensionless ratio $\Delta x/L_{r}$. The state at the end of region *r* is related to the state at its start by

(5) $$s_{r}\left(\ell_{r}\right)=M_{r}\left(\ell_{r}\right)s_{r}\left(0\right)+b_{r},$$

where $M_{r}\left(\ell_{r}\right)$ propagates the input state across the complete region and $b_{r}$ is the source vector associated with carrier generation inside that region.

A carrier contribution generated at the local position $x^{\prime }$ must travel the remaining distance $\ell_{r}-x^{\prime }$ before reaching the end of the region. The source vector is therefore

(6) $$b_{r}=\int_{0}^{\ell_{r}}M_{r}\left(\ell_{r}-x^{\prime }\right)\left(\begin{array}{c} 0\\ -g_{r}\left(x^{\prime }\right) \end{array}\right)\;dx^{\prime }.$$

Equations (5) and (6) use the same matrix function with different propagation distances. The matrix $M_{r}\left(\ell_{r}\right)$ acts across the complete region. Inside the source integral, $M_{r}\left(\ell_{r}-x^{\prime }\right)$ propagates only the contribution generated at $x^{\prime }$ through the remaining part of the region. A contribution generated at $x^{\prime }=0$ travels across the complete region. A contribution generated at $x^{\prime }=\ell_{r}$ is already at the end of the region and is multiplied by $M_{r}\left(0\right)=I$, where *I* is the 2×2 identity matrix.

At the direct base–BSF interface, the normalized excess concentration and the scaled diffusion flux are continuous:

(7) $$u_{B}\left(W_{B}\right)=u_{S}\left(0\right),\;G_{B}\left(W_{B}\right)=G_{S}\left(0\right).$$

The flux condition expresses carrier conservation across the interface. The emitter–base depletion region is not treated as a direct quasi-neutral interface. Its injection conditions are introduced in the next subsection. Recombination at the external surfaces is also imposed there through Robin boundary conditions.

For tabulated optical data, Equation (6) is evaluated by trapezoidal quadrature. The three matrix–vector pairs $\left(M_{E},b_{E}\right)$, $\left(M_{B},b_{B}\right)$, and $\left(M_{S},b_{S}\right)$ are calculated before the voltage sweep. Repeated forward evaluations then require only small matrix operations and the device-level boundary closure.

### 2.2. Device-Level Formulation and Verification

We next describe the semiconductor model used for benchmarking and calibration. Under low injection in quasi-neutral regions, minority-carrier transport is governed by Equation (1) with g(x) the generation profile and 1/τ an effective recombination rate. The normalization $c_{0}$ is chosen as the equilibrium minority concentration: $c_{0}=n_{0}$ for minority electrons in *p*-type material and $c_{0}=p_{0}$ for minority holes in *n*-type material.

With this scaling, *u* is a normalized excess minority concentration, and $G=Dc_{0}u^{\prime }$ is proportional to the physical diffusion flux. In semiconductor language, the diode current is obtained from depletion-edge fluxes (Section 2.2), and surface recombination is naturally expressed as a Robin constraint in (u,G).

If illumination is represented by a distributed generation rate g(x), the TM formulation uses g(x) directly through the source vectors $b_{r}$. In the special ideal-collection approximation, the optical input may be condensed into the scalar current density

(8) $$J_{\mathrm{ph}}^{\mathrm{ideal}}=q\int_{\Omega }g\left(x\right)\;dx,$$

where $J_{\mathrm{ph}}^{\mathrm{ideal}}$ is the ideal photogenerated current density, *q* is the elementary charge, and $\Omega =[0,d_{0}]$ is the complete device domain. This condensation is a model choice and is not required by the TM core.

We first verify that the transfer-matrix (TM) formulation reproduces the classical two-layer $p^{+}/n$ model under dark conditions (g=0). In this case, all source vectors vanish and each transport region is described only by its homogeneous transfer matrix.

Consider a front $p^{+}$ quasi-neutral region of thickness $W_{p}$ and a rear *n* quasi-neutral region of thickness $W_{n}$. The depletion region is not resolved. Instead, the normalized injection at both depletion edges is prescribed as

(9) $$u_{p}\left(W_{p};V_{d}\right)=u_{0}\left(V_{d}\right),\;u_{n}\left(0;V_{d}\right)=u_{0}\left(V_{d}\right),$$

where $V_{d}$ is the internal diode voltage and $u_{0}\left(V_{d}\right)$ is the normalized depletion-edge injection. Under the ideal low-injection relation, $u_{0}\left(V_{d}\right)=\exp\left(V_{d}/V_{T}\right)-1$, where $V_{T}=k_{B}T/q$ is the thermal voltage, $k_{B}$ is the Boltzmann constant, and *T* is the absolute temperature.

At the front surface (in $p^{+}$), minority electrons experience an effective surface recombination velocity $S_{n}$, leading to the Robin condition

(10) $$G_{p}\left(0\right)=\gamma_{F}u_{p}\left(0\right),\;\gamma_{F}=S_{n}c_{0,p}.$$

At the rear surface (in *n*), minority holes experience $S_{p}$,

(11) $$G_{n}\left(W_{n}\right)=-\gamma_{R}u_{n}\left(W_{n}\right),\;\gamma_{R}=S_{p}c_{0,n}.$$

Here, $S_{n}$ and $S_{p}$ are effective boundary parameters. They represent the net recombination losses associated with the front and rear surfaces and depend on both the interface properties and the local doping conditions near the surface [31,32].

Eliminating the boundary states using the homogeneous layer matrices and Equations (10) and (11) yields the classical saturation current density

(12) $$J_{0,\mathrm{pn}}=q\left[\frac{D_{n}n_{i}^{2}}{L_{n}N_{A}}\;F_{F}\left(W_{p},L_{n},D_{n},S_{n}\right)+\frac{D_{p}n_{i}^{2}}{L_{p}N_{D}}\;F_{R}\left(W_{n},L_{p},D_{p},S_{p}\right)\right],$$

with

(13) $$\begin{array}{cc} F_{F} & =\frac{\cosh\left(W_{p}/L_{n}\right)+\left(D_{n}/\left(S_{n}L_{n}\right)\right)\sinh\left(W_{p}/L_{n}\right)}{\left(D_{n}/\left(S_{n}L_{n}\right)\right)\cosh\left(W_{p}/L_{n}\right)+\sinh\left(W_{p}/L_{n}\right)},\\ F_{R} & =\frac{\cosh\left(W_{n}/L_{p}\right)+\left(D_{p}/\left(S_{p}L_{p}\right)\right)\sinh\left(W_{n}/L_{p}\right)}{\left(D_{p}/\left(S_{p}L_{p}\right)\right)\cosh\left(W_{n}/L_{p}\right)+\sinh\left(W_{n}/L_{p}\right)}. \end{array}$$

The classical hyperbolic factors therefore follow directly from the same two-layer transfer-matrix formulation.

We next consider a three-region $p^{+}$–*n*–$n^{+}$ structure: emitter (*E*), base (*B*), and BSF (*S*). Each region contributes a matrix–vector pair $\left(M_{r},b_{r}\right)$ through Equation (5).

In the $p^{+}/n$ model, the rear boundary loss $S_{p}$ acts directly on the *n* base boundary. In contrast, in the $p^{+}$–*n*–$n^{+}$ model, the rear metallization is separated from the base by an explicit $n^{+}$ region (BSF). The rear parameter $S_{\mathrm{rear}}$ therefore acts on minority holes in the *BSF*, and its influence on the base is filtered by transport through the BSF. Consequently, numerical values of “rear SRV” are not expected to be directly transferable between the two models because they represent different effective physics.

Continuity at the base–BSF interface yields a combined transfer relation from the base-side depletion edge to the rear surface:

(14) $$s_{S}\left(\ell_{S}\right)=M_{SB}\;s_{B}\left(0\right)+b_{SB},\;M_{SB}=M_{S}M_{B},\;b_{SB}=M_{S}b_{B}+b_{S}.$$

The rear Robin constraint is imposed at $x=\ell_{S}$ on the BSF state (analog of (11) but on the BSF variables).

Current and voltage notation. The TM closure provides the minority-carrier fluxes at the emitter-side and base-side depletion edges. We distinguish the intrinsic semiconductor response from the external terminal response as follows.

The symbol $V_{d}$ denotes the internal diode voltage applied to the diffusion–reaction core. The subscript *d* in $V_{d}$ means diode; it does not denote a separate dark voltage. The positive dark, or diode-injection, current density is denoted by $J_{d}\left(V_{d}\right)$ and is calculated from the same TM model after setting the optical generation to zero, g(x)=0. Equivalently, all layer source vectors are set to zero. With the photovoltaic sign convention,

(15) $$J_{d}\left(V_{d}\right)=q\;G_{E}^{\mathrm{dark}}\left(\ell_{E};V_{d}\right)-q\;G_{B}^{\mathrm{dark}}\left(0;V_{d}\right),$$

where $G_{E}^{\mathrm{dark}}\left(\ell_{E};V_{d}\right)$ and $G_{B}^{\mathrm{dark}}\left(0;V_{d}\right)$ are the dark scaled fluxes at the emitter-side and base-side depletion edges, respectively, and $\ell_{E}=W_{E}$ is the emitter thickness. The current $J_{d}$ is positive under forward diode injection. Although Equation (15) contains only the two depletion-edge fluxes, the rear-field contribution is included in $G_{B}^{\mathrm{dark}}\left(0;V_{d}\right)$. This flux is obtained from the combined base–rear-field block, $M_{SB}=M_{S}M_{B}$, together with the rear-surface boundary condition. Therefore, the BSF affects the dark current through its thickness, transport parameters, and rear recombination condition. No separate BSF current term is required because the $n/n^{+}$ rear field is not treated as an independent rectifying junction.

Under illumination, the intrinsic current density calculated by the semiconductor transport model is denoted by $J_{\mathrm{core}}\left(V_{d};g\right)$. In this notation, the semicolon separates the internal voltage $V_{d}$ from the prescribed generation profile g(x). The core current includes the distributed optical source through the vectors $b_{r}$ and the voltage-driven injection through $u_{0}\left(V_{d}\right)$, but it does not include the external series or shunt losses. Under the linear low-injection assumptions used here,

(16) $$J_{\mathrm{core}}\left(V_{d};g\right)=J_{\mathrm{ph},\mathrm{TM}}\left(g\right)-J_{d}\left(V_{d}\right),$$

where $J_{\mathrm{ph},\mathrm{TM}}\left(g\right)$ is the source-driven current collected by the TM model. It is defined by

(17) $$J_{\mathrm{ph},\mathrm{TM}}\left(g\right)=J_{\mathrm{core}}\left(0;g\right).$$

In general, $J_{\mathrm{ph},\mathrm{TM}}\left(g\right)$ differs from the ideal optical current $J_{\mathrm{ph}}^{\mathrm{ideal}}$ in Equation (8), because the TM value includes transport and recombination losses. In the MATLAB R2024b Update 3 (24.2.0.2806996, 64-bit) implementation, $J_{\mathrm{core}}$ is evaluated directly from the complete source vectors and the injection condition; separate illuminated and dark solver calls are not required.

The external current density is denoted by $J_{\mathrm{term}}$, and the voltage measured between the external contacts is denoted by $V_{\mathrm{term}}$. The area-normalized series resistance is $R_{s}$, and the area-normalized shunt resistance is $R_{sh}$; both are expressed in $\Omega \;{\mathrm{cm}}^{2}$. The terminal current and voltage are obtained parametrically from the internal diode voltage:

(18) $$J_{\mathrm{term}}\left(V_{d};g\right)=J_{\mathrm{core}}\left(V_{d};g\right)-\frac{V_{d}}{R_{sh}},\;V_{\mathrm{term}}\left(V_{d};g\right)=V_{d}-R_{s}J_{\mathrm{term}}\left(V_{d};g\right).$$

Equivalently, $V_{d}=V_{\mathrm{term}}+R_{s}J_{\mathrm{term}}$. When lumped electrical losses are disabled, $R_{s}=0$ and $R_{sh}\to \infty$, so that $J_{\mathrm{term}}=J_{\mathrm{core}}$ and $V_{\mathrm{term}}=V_{d}$. Unless otherwise stated, the reported current–voltage curves and photovoltaic metrics use the terminal quantities $J_{\mathrm{term}}$ and $V_{\mathrm{term}}$.

The experimental reference cell is calibrated in two stages. First, the optical-generation scale factor $C_{g}$ and a common effective surface-recombination velocity, $S_{\mathrm{eff}}=S_{\mathrm{front}}=S_{\mathrm{rear}}$, are fitted simultaneously to the measured $J_{sc}$ and $V_{oc}$. During this stage, the SRH lifetime and shunt resistance are kept at their reference values, $\tau_{\mathrm{SRH}}=1.5\;\mathrm{ms}$ and $R_{sh}=2.0\times 10^{4}\;\Omega \;{\mathrm{cm}}^{2}$.

In the second stage, $C_{g}$ and $S_{\mathrm{eff}}$ are fixed, and the non-geometrical series-resistance contribution $R_{s,\mathrm{other}}$ is fitted to the measured fill factor. No transport or recombination parameter is changed during this second stage.

### 2.3. Optical Generation Model and Input Data

The transfer-matrix formulation developed in this paper describes minority-carrier transport through the semiconductor regions and is not an electromagnetic transfer-matrix treatment of the complete optical stack. The optical source is therefore calculated separately before the electrical transport problem is solved. In this sequential optical–electrical approach, spectral data are first converted into a depth-dependent generation profile and then used as input to the carrier-transport model, as commonly performed in photovoltaic device simulation [33].

More generally, the front-reflectance spectrum required by the optical source model may be supplied either from experimental measurements or from an independent optical calculation, such as a multilayer optical transfer-matrix model when the optical stack and its material parameters are sufficiently characterized. In this paper, the experimentally measured total front reflectance of the metallized n-PERT reference cell reported in Ref. [24] is used. This spectrum was measured using a Perkin Elmer 950 spectrophotometer and includes the optical response of the actual textured and antireflection-coated front surface. The reference cell has a random-pyramid textured silicon surface and a front boron silicate glass/silicon nitride (BSG/SiN_*x*_) passivation/antireflection structure; consequently, the measured reflectance includes both specular and diffuse contributions.

The remaining optical inputs are the AM1.5G spectral irradiance [34] and the optical constants of crystalline silicon [35]. The reflectance R(λ) used here therefore represents the measured total front optical response of the reference device and should not be interpreted as the Fresnel reflectance of an uncoated planar silicon wafer. The measured spectrum exhibits a pronounced minimum near 560 nm, consistent with the antireflection behavior of the reference-cell front surface, and remains low over a broad part of the visible and near-infrared spectral range.

When the extinction coefficient is used, the absorption coefficient is calculated as α(λ)=4πk(λ)/λ. Thus, k(λ) determines the attenuation of radiation after it enters the silicon, whereas the experimentally measured R(λ) specifies the fraction of incident radiation reflected at the front optical boundary. The incident spectral irradiance is converted into photon flux through $\Phi_{\lambda }\left(\lambda \right)=\lambda I_{\lambda }\left(\lambda \right)/\left(hc\right)$.

For front-side illumination, the depth-dependent generation rate is

(19) $$g\left(x\right)=\int_{\lambda_{\min}}^{\lambda_{\max}}\left[1-R(\lambda )\right]\alpha \left(\lambda \right)\Phi_{\lambda }\left(\lambda \right)\exp\left[-\alpha (\lambda )x\right]\;d\lambda .$$

Within the present application of this reduced single-pass optical description, the measured R(λ) spectrum is used as the front optical input. The fractions of the incident optical power transmitted through and absorbed within a silicon wafer of thickness $d_{0}$ are therefore

(20) $$T\left(\lambda \right)=\left[1-R(\lambda )\right]\exp\left[-\alpha \left(\lambda \right)d_{0}\right],$$

and

(21) $$A\left(\lambda \right)=\left[1-R(\lambda )\right]\left\{1-\exp\left[-\alpha \left(\lambda \right)d_{0}\right]\right\},$$

respectively, so that R(λ)+T(λ)+A(λ)=1. In spectral regions where $\alpha \left(\lambda \right)d_{0}\gg 1$, the transmittance becomes negligible and A(λ)≃1−R(λ). Therefore, a very small front reflectance in an optically thick silicon wafer corresponds to strong absorption and is fully consistent with energy conservation. The wavelength integral in Equation (19) is calculated from tabulated curves. The resulting g(x) profile is stored on a front-clustered nonuniform spatial grid, $x=d_{\max}t^{3}$ with 0≤t≤1, and repartitioned among the emitter, base, and rear-field regions for each device geometry. A 2501-point master grid extending to $d_{\max}=250$ µm is used so that the 160, 180, and 200 µm scenarios share the same optical source. Convergence was verified by comparing q∫g(x)dx with the direct Beer–Lambert depth integral for each prescribed thickness; the relative discrepancy was below 0.001% in all three cases.

**Figure 1:**
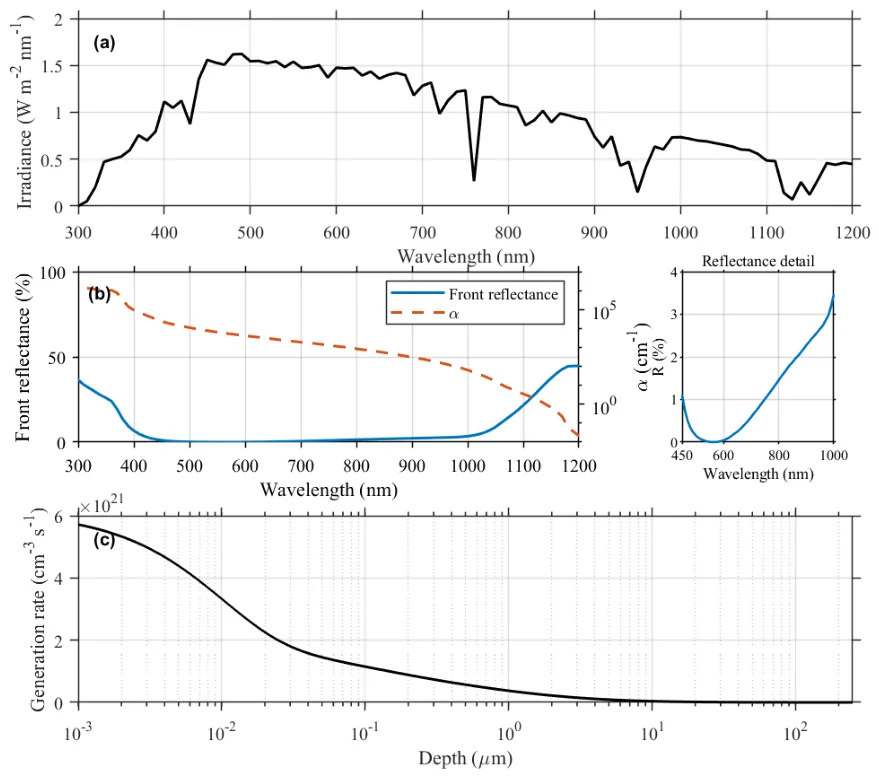
Optical inputs and calculated depth-dependent generation rate. The final panel provides the source term used by the transfer-matrix model. (**a**) AM1.5G spectral irradiance; (**b**) front reflectance and silicon absorption coefficient α, with an inset showing the reflectance detail; and (**c**) calculated generation rate g(x).

Figure 1 summarizes the optical inputs and the calculated generation profile used by the electrical transfer-matrix solver.

### 2.4. Reference n-PERT Architecture and Diffusion Profiles

The reference device is based on the experimental bifacial n-PERT solar cell reported in a previous work, where a one-dimensional drift–diffusion model implemented in COMSOL Multiphysics was compared with the reported experimental photovoltaic figures of merit and used for genetic-algorithm optimization of the cell geometry and doping parameters [24]. In this paper, the same experimental device provides the reference geometry and electrical operating point for the calibration of the transfer-matrix model.

The experimental device provides the reference geometry and photovoltaic operating point used to calibrate the transfer-matrix model.

**Figure 2:**
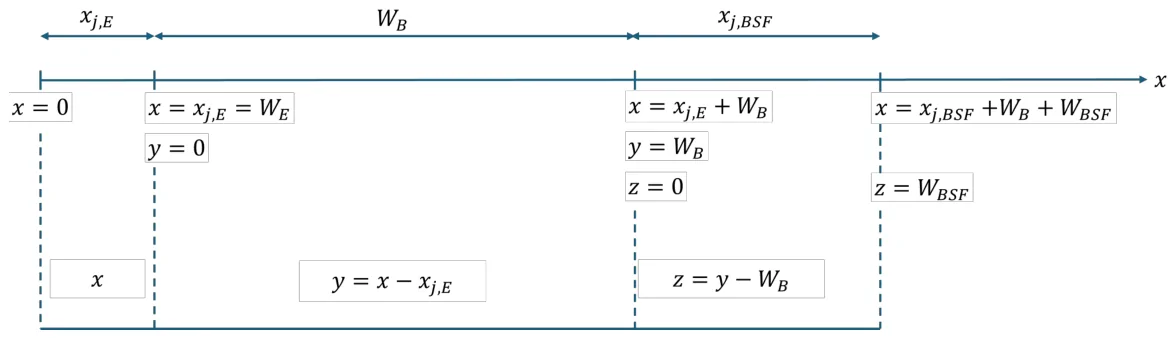
One-dimensional n-PERT cell structure and coordinate definitions. The front emitter occupies $0\leq x\leq W_{E}$, the base occupies $0\leq y\leq W_{B}$, and the rear-field coordinate is $0\leq z\leq W_{\mathrm{BSF}}$. The horizontal arrow indicates the increasing global depth coordinate from the front to the rear surface, while the dashed vertical lines indicate the device boundaries and region interfaces defining the corresponding local-coordinate limits.

The device contains a front $p^{+}$ emitter, an *n*-type base, and a rear $n^{+}$ field. Figure 2 defines the global coordinate *x* and the local coordinates used in the base and rear-field regions. The reference geometry, dopant concentrations, surface recombination velocities, contact dimensions, and electrical parameters are used in the forward benchmarks and calibration stage. The optimization bounds are defined separately because they describe the design space rather than the reference cell.

The optimization model describes the front emitter and the rear field through complementary-error-function profiles, corresponding to the ideal constant-source solution of Fick’s second law for thermal dopant diffusion in silicon [18,28]. This representation provides a physically motivated approximation of the diffused regions while keeping the model suitable for repeated optimization.

(22) $$N_{A}\left(x\right)=N_{A,s}\mathrm{erfc}\left(\frac{x}{2L_{\mathrm{diff},E}}\right).$$

The emitter depth is set by $N_{A}\left(W_{E}\right)=N_{D,B}$ and is given by

(23) $$W_{E}=2L_{\mathrm{diff},E}{\mathrm{erfc}}^{-1}\left(\frac{N_{D,B}}{N_{A,s}}\right).$$

The rear $n^{+}$ region is described as

(24) $$N_{D}\left(z\right)=N_{D,s}^{\mathrm{rear}}\mathrm{erfc}\left(\frac{z}{2L_{\mathrm{diff},R}}\right),$$

with $N_{D}\left(W_{\mathrm{BSF}}\right)=N_{D,B}$. Therefore,

(25) $$W_{\mathrm{BSF}}=2L_{\mathrm{diff},R}{\mathrm{erfc}}^{-1}\left(\frac{N_{D,B}}{N_{D,s}^{\mathrm{rear}}}\right).$$

The base thickness is $W_{B}=d_{0}-W_{E}-W_{\mathrm{BSF}}$.

The transfer-matrix model uses the thickness-averaged concentrations

(26) $$N_{A,E}^{\mathrm{eff}}=\frac{1}{W_{E}}\int_{0}^{W_{E}}N_{A}\left(x\right)\;dx,\;N_{D,\mathrm{BSF}}^{\mathrm{eff}}=\frac{1}{W_{\mathrm{BSF}}}\int_{0}^{W_{\mathrm{BSF}}}N_{D}\left(z\right)\;dz.$$

These values are used for mobility, equilibrium carrier densities, Auger recombination, band-gap narrowing, and the layer matrices. The sheet resistances are obtained from the complete conductivity profiles.

### 2.5. Doping-Dependent Transport and Electrical Losses

Carrier mobilities are evaluated with the Arora model [36]. High-doping effects are included through Slotboom band-gap narrowing [37,38] and dopant-dependent Auger recombination [39]. The effective minority-carrier lifetimes are

(27) $$\frac{1}{\tau_{n,E}^{\mathrm{eff}}}=\frac{1}{\tau_{n,E}^{\mathrm{SRH}}}+C_{p}{\left(N_{A,E}^{\mathrm{eff}}\right)}^{2},\;\frac{1}{\tau_{p,j}^{\mathrm{eff}}}=\frac{1}{\tau_{p,j}^{\mathrm{SRH}}}+C_{n}{\left(N_{D,j}^{\mathrm{eff}}\right)}^{2},$$

where j∈{B,S} denotes the base or BSF region. These terms provide a physical cost for excessive doping and avoid an empirical penalty on the design variables.

The front and rear electrical losses are represented by the total area-normalized series resistance

(28) $$R_{s}=R_{s,\mathrm{other}}+R_{s,E}+R_{c,E}+R_{s,R}+R_{c,R}.$$

Here, $R_{s,\mathrm{other}}$ represents the lumped series-resistance contributions that are not explicitly resolved by the reduced model. The terms $R_{s,E}$ and $R_{s,R}$ represent the lateral current-transport losses in the front emitter and rear-field regions, respectively, whereas $R_{c,E}$ and $R_{c,R}$ represent the corresponding front and rear contact resistance contributions. All terms in Equation (28) are area-normalized and are expressed in $\Omega \;{\mathrm{cm}}^{2}$. The contribution $R_{s,\mathrm{other}}$ is obtained during calibration, while the remaining terms depend on the diffusion profiles and contact geometry.

The emitter and rear-field sheet resistances are calculated from the complete diffusion profiles,

(29) $$R_{\mathrm{sh},E}={\left[q\int_{0}^{W_{E}}\mu_{p}\left(N_{A}\right)N_{A}\left(x\right)\;dx\right]}^{-1},\;R_{\mathrm{sh},R}={\left[q\int_{0}^{W_{\mathrm{BSF}}}\mu_{n}\left(N_{D}\right)N_{D}\left(z\right)\;dz\right]}^{-1}.$$

For the parallel-finger geometry, $s_{E}$ and $s_{R}$ denote the front and rear finger pitches, respectively, which are defined as the center-to-center distances between adjacent fingers. The subscripts *E* and *R* refer to the emitter and rear-field sides. The lateral sheet-resistance contributions are obtained by averaging the distributed Joule losses over one finger pitch. Assuming uniform current injection, symmetric collection, and finger widths much smaller than the corresponding pitches, the area-normalized contributions are approximated as

(30) $$R_{s,E}=R_{\mathrm{sh},E}\frac{s_{E}^{2}}{12},\;R_{s,R}=R_{\mathrm{sh},R}\frac{s_{R}^{2}}{12}.$$

The factor 1/12 results from the integration of the squared lateral sheet current between adjacent parallel fingers [40].

Specific contact resistivity generally decreases with increasing near-surface doping, although its exact dependence also varies with the metallization process and the properties of the metal–semiconductor interface [41,42]. To represent this trend within the reduced electrical-loss model, bounded semi-empirical power-law relations are used:

(31) $$\rho_{c,E}=\rho_{c,E}^{\mathrm{ref}}{\left(\frac{N_{A,s}}{N_{E}^{\mathrm{ref}}}\right)}^{-m_{E}},\;\rho_{c,R}=\rho_{c,R}^{\mathrm{ref}}{\left(\frac{N_{D,s}^{\mathrm{rear}}}{N_{R}^{\mathrm{ref}}}\right)}^{-m_{R}}.$$

Here, $\rho_{c}^{\mathrm{ref}}$, $N^{\mathrm{ref}}$, and *m* are contact-model parameters. The calculated contact resistivities are restricted to the lower and upper bounds listed in Table A2.

For finger widths $w_{E}$ and $w_{R}$, the contacted fractions are $f_{E}=w_{E}/s_{E}$ and $f_{R}=w_{R}/s_{R}$. Thus, $R_{c,E}=\rho_{c,E}/f_{E}$ and $R_{c,R}=\rho_{c,R}/f_{R}$ [40]. Only front-side illumination is considered. The rear grid affects electrical collection, but no rear optical source is added to g(x).

The model combines newly assembled elements with standard constitutive relations from the literature. The transfer-matrix formulation used here, including the regional carrier-state representation, the source-vector treatment of distributed generation, the explicit emitter–base–rear-field coupling, and the device-level boundary closure, is derived in Section 2.1 and Section 2.2 for the present $p^{+}$–*n*–$n^{+}$ structure. The physics-constrained optimization framework also links the diffused-region profiles, derived junction depths, transport properties, surface recombination, and resistive losses within each candidate evaluation.

By contrast, the underlying material and transport relations are established models adopted from the literature. These include the Arora mobility model, band-gap narrowing, Auger recombination, the complementary-error-function description of constant-source dopant diffusion, and the standard expressions used for sheet and contact resistance. The contribution of this paper is therefore the integration of these relations into the reduced-order transfer-matrix and inverse-design framework described above. No new intrinsic silicon material properties are measured or extracted in this paper; the new model outputs are the optimized doping concentrations, the derived junction depths, the associated transport and loss balances, and the device performance predicted by the calibrated framework.

### 2.6. Calibration and Sensitivity Analysis

The experimental reference cell was calibrated in two stages after the transport, optical, and electrical-loss models had been fully specified. First, the optical-generation scale factor $C_{g}$ and a common effective surface-recombination velocity, $S_{\mathrm{eff}}=S_{\mathrm{front}}=S_{\mathrm{rear}}$, were fitted simultaneously to the reported $J_{sc}$ and $V_{oc}$ of the reference cell. During this stage, the SRH lifetime and shunt resistance were kept at $\tau_{\mathrm{SRH}}=1.5$ ms and $R_{sh}=2.0\times 10^{4}\;\Omega \;{\mathrm{cm}}^{2}$, respectively.

In the second stage, $C_{g}$ and $S_{\mathrm{eff}}$ were kept fixed, and the non-geometrical series-resistance contribution $R_{s,\mathrm{other}}$, defined in Equation (28), was fitted to the reported fill factor. No transport or recombination parameter was changed during this second stage.

To examine the local sensitivity of the calibration, $C_{g}$ and $S_{\mathrm{eff}}$ were subsequently varied independently around the calibrated solution while the remaining parameters were kept fixed. For each parameter pair, $J_{sc}$ and $V_{oc}$ were recalculated and compared with the reported experimental values. Using the reported uncertainties $\sigma_{J_{sc}}=0.03$ mA/cm^2^ and $\sigma_{V_{oc}}=2$ mV, the normalized mismatch was defined as

$$\chi^{2}={\left(\frac{J_{sc}-J_{sc,\exp}}{\sigma_{J_{sc}}}\right)}^{2}+{\left(\frac{V_{oc}-V_{oc,\exp}}{\sigma_{V_{oc}}}\right)}^{2}.$$

For this diagnostic analysis, the reported uncertainties were treated as independent standard uncertainties. Under this interpretation, $\chi^{2}=2.30$ and $\chi^{2}=5.99$ define nominal 68% and 95% joint regions, respectively, for the two fitted parameters. The sensitivity of the second calibration stage was examined separately by varying $R_{s,\mathrm{other}}$ while keeping $C_{g}$ and $S_{\mathrm{eff}}$ fixed and comparing the resulting fill factor with the reported value FF=0.783±0.002.

## 3. Physics-Constrained Global Optimization

### 3.1. Fixed-Thickness Design Scenarios and Objective Function

The optimization is formulated for prescribed wafer-thickness scenarios. For each selected value of $d_{0}$, the independent design vector is

(32) $$\theta =\left(N_{A,s},N_{D,B},N_{D,s}^{\mathrm{rear}}\right).$$

The emitter and rear-field depths follow from Equations (23) and (25). The genetic algorithm maximizes the terminal power-conversion efficiency, which is defined as

(33) $$\eta_{\mathrm{terminal}}=\frac{P_{\mathrm{mpp},\mathrm{terminal}}}{P_{\mathrm{in}}},$$

where $P_{\mathrm{mpp},\mathrm{terminal}}$ is the maximum terminal electrical power density and $P_{\mathrm{in}}$ is the incident optical power density.

The comparison considers prescribed wafer thicknesses of $d_{0}=160$, 180, and 200 µm. The 180 µm case corresponds to the experimentally calibrated reference geometry, whereas the 160 and 200 µm cases provide thinner and thicker geometrical constraints for testing the robustness of the optimization framework. These cases are not used to infer an optimal industrial thickness. For each prescribed thickness, the optical generation profile g(x) is restricted to the corresponding device domain and then retained as the prescribed optical input throughout that optimization scenario. For every candidate design θ, however, the derived layer boundaries and doping-dependent transport parameters are updated. Consequently, the regional matrices $M_{r}\left(\theta \right)$, source vectors $b_{r}\left(\theta ;g\right)$, and collected photocurrent $J_{\mathrm{ph},\mathrm{TM}}\left(\theta ;g\right)$ are recomputed before evaluating the terminal current–voltage curve of that candidate.

This scenario-based formulation avoids treating wafer thickness as a free optimization variable within the present optical model. The purpose is to compare how the calibrated physics-constrained optimization responds to fixed geometrical assumptions rather than to claim that the simplified optical treatment determines the technological optimum wafer thickness.

### 3.2. Search Strategy

A hybrid physics-constrained genetic algorithm–sequential quadratic programming (GA–SQP) approach was used. MATLAB’s genetic algorithm first explored the design space, and the best feasible solution was then refined with fmincon using the sequential quadratic programming algorithm. The same parameter bounds and numerical budget were applied to all prescribed-thickness scenarios. For reproducibility, the random-number generator was initialized with the Mersenne Twister algorithm and seed 1 using rng(1,’twister’), and the calibrated reference design was included in the initial GA population. The complete search settings, tolerances, and parameter bounds are listed in Table A3.

### 3.3. Numerical Implementation

The numerical implementation follows directly from the matrix–vector transfer structure:

(i) partition g(x) per layer and precompute $\left\{M_{r},b_{r}\right\}$ (quadrature for $b_{r}$ if g≠0);

(ii) for each diode voltage $V_{d}$, evaluate the junction injection law $u_{0}\left(V_{d}\right)$ and enforce the linear boundary/interface constraints (e.g., Robin surface conditions and depletion-edge relations), which reduces to a small algebraic closure in (u,G);

(iii) assemble the candidate-specific intrinsic current density $J_{\mathrm{core}}\left(V_{d};\theta ,g\right)$ directly from the depletion-edge fluxes in the TM model or through the reduced superposition form in the textbook PN model. For resistive cases, the intrinsic response is first evaluated on a grid of internal diode voltages $V_{d}$. The terminal current density $J_{\mathrm{term}}$ and terminal voltage $V_{\mathrm{term}}$ are then obtained from Equation (18). This parametric representation avoids a separate nonlinear root calculation at each terminal voltage.

The matrices and source vectors are therefore constructed once per candidate design, before its internal-voltage sweep, rather than once for the complete optimization run. For a fixed candidate, $b_{r}\left(\theta ;g\right)$ and $J_{\mathrm{ph},\mathrm{TM}}\left(\theta ;g\right)$ remain unchanged during the sweep over $V_{d}$. When the optimizer proposes a new θ, the layer geometry, transport parameters, matrices, source vectors, and collected photocurrent are recomputed. After this candidate-specific precomputation, each current–voltage curve requires only small matrix operations and algebraic post-processing, making the formulation suitable for global optimization and inverse design.

After optimization, the change in efficiency was further analyzed through an interaction-aware loss decomposition. Five mechanisms were considered explicitly: emitter/rear-field sheet resistance, front/rear contact resistance, Auger recombination, band-gap narrowing, and front/rear surface recombination. For each baseline and optimized design, all $2^{5}$ combinations of these mechanisms were evaluated while retaining the remaining transport model, $R_{s,\mathrm{other}}$, $R_{sh}$, and SRH recombination. The marginal contribution of each mechanism was then averaged over all possible orders of inclusion. This permutation-averaged procedure distributes nonlinear interactions among the five mechanisms instead of assigning the result to an arbitrary sequential removal order.

The corresponding change in efficiency can therefore be written as the sum of the changes associated with the five evaluated mechanisms and a remaining design/transport contribution. The latter represents the change in carrier transport and collection that remains when the five selected loss mechanisms are disabled. The complete numerical decomposition and the corresponding MATLAB implementation are provided in the Supplementary Materials.

## 4. Results

### 4.1. Forward Benchmarks in Limiting Regimes

The transfer-matrix solver was first evaluated in controlled limiting regimes using a uniform generation rate,

(34) $$g\left(x\right)=g_{0}\;\mathrm{in}\;\mathrm{the}\;\mathrm{photoactive}\;\mathrm{regions}.$$

This source isolates bulk transport, boundary recombination, and geometric effects before a spatially varying optical profile is introduced. Two models are compared: (i) the classical two-region $p^{+}/n$ formulation (PN), and (ii) the three-region $p^{+}\left|n\right|n^{+}$ formulation (PNN), in which the BSF region is explicitly included. No lumped series or shunt losses are included in this benchmark. Therefore, $J_{\mathrm{term}}=J_{\mathrm{core}}$ and $V_{\mathrm{term}}=V_{d}$. Both models use the photovoltaic sign convention with the short-circuit current density $J_{sc}=J_{\mathrm{term}}\left(V_{\mathrm{term}}=0\right)>0$ under illumination.

The four cases are outlined below:

- Case A (baseline): nominal surface recombination velocities $\left(S_{n},S_{p}\right)$ and nominal geometry.
- Case B ($S_{n},S_{p}\to \infty$): strongly recombinative boundary limit.
- Case C ($S_{n},S_{p}=10^{6}$ cm/s): large but finite surface recombination, which is used to verify convergence to Case B.
- Case D (thin base): reduced base thickness $W_{B}=60\;\mu m$, which is used to test geometric sensitivity and the role of the explicit BSF region.

**Figure 3:**
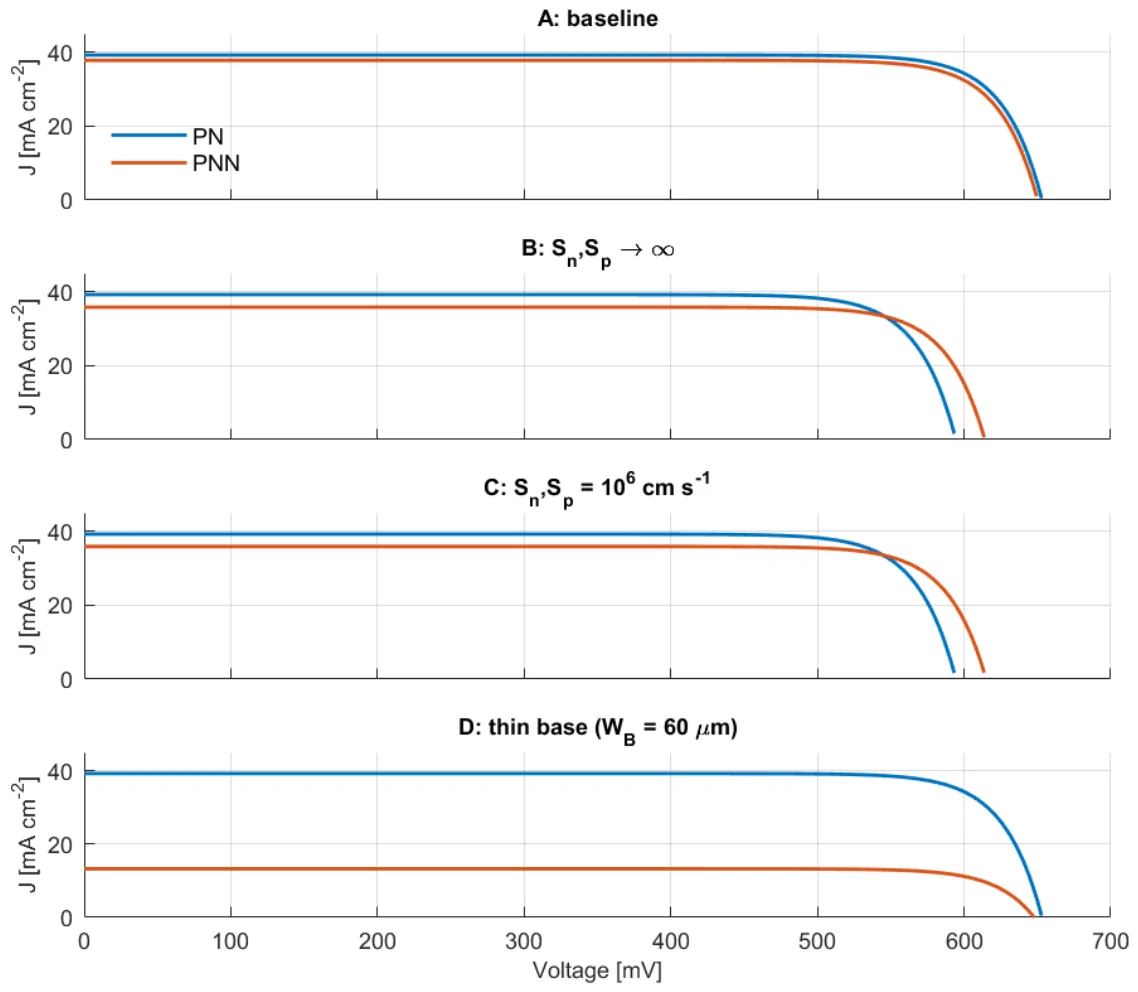
Forward benchmarks under constant generation $g\left(x\right)=g_{0}$: comparison of PN and PNN *J*–*V* curves for Cases A–D.

Figure 3 shows the calculated *J*–*V* curves, and Table 1 reports the short-circuit current density $J_{sc}$, the open-circuit voltage $V_{oc}$, and the fill factor (FF).

The main trends are summarized below.

(i) Surface-recombination-dominated limit in PN. Cases B and C reduce $V_{oc}$ and FF. Their nearly identical results confirm that $S_{n},S_{p}=10^{6}\;\mathrm{cm}/s$ is a good numerical approximation to the S→∞ limit for the present parameter range [43].

(ii) PNN response under high surface recombination. The PNN model also shows lower collected current and voltage in Cases B and C. Unlike the PN reduction, it calculates collection through the emitter, base, and rear field; therefore, high boundary losses can also reduce $J_{sc}$. The explicit rear field changes how rear losses are transmitted to the base instead of representing them only through an effective rear boundary condition [44].

(iii) Geometric sensitivity and role of the explicit BSF layer. In Case D, the PN result remains unchanged by construction, whereas the PNN current decreases strongly when the base and its generation domain are shortened. The lower current is expected because $g_{0}$ retains the normalization of the 180 μm reference device. This case shows the geometric sensitivity provided by the explicit base and rear-field regions. Wafer thickness is also known to affect the loss mechanisms of n-type crystalline-silicon cells [45], but the PN–PNN difference reported here follows from the different model structures.

Overall, the benchmarks confirm stable behavior in boundary-dominated and geometry-perturbed cases. The next sections replace the constant source with a tabulated generation profile and then introduce series and shunt losses.

### 4.2. Forward Benchmark with Tabulated Generation Profiles

The same reference geometry and surface-recombination parameters used in the constant-generation benchmarks of Section 4.1 are retained. For the PNN calculation, a common SRH lifetime of 265 µs is used in the three transport regions. The uniform source $g_{0}$ is replaced by a tabulated profile g(x) defined on a global one-dimensional mesh covering the complete device thickness $d_{0}$. Within each quasi-neutral region, the profile is restricted to the local subdomain and incorporated through the layer relation in Equation (5). The source vector $b_{r}$ is calculated by numerical quadrature of Equation (6). No additional fitting or tuning is introduced at this stage. Lumped electrical losses are also disabled, so $J_{\mathrm{term}}=J_{\mathrm{core}}$ and $V_{\mathrm{term}}=V_{d}$.

For reference, the ideal photogenerated current density is evaluated from Equation (8) over the complete device domain $\Omega =[0,d_{0}]$. This value represents the perfect-collection limit and is not imposed as an input to the TM model. For the considered profile,

$$J_{\mathrm{ph}}^{\mathrm{ideal}}=38.1240\;\mathrm{mA}/{\mathrm{cm}}^{2}.$$

Using the same tabulated generation profile, the illuminated *J*–*V* curves are computed with both models:

- PN (classical $p^{+}/n$ model): Illumination enters only through the scalar quantity $J_{\mathrm{ph}}^{\mathrm{ideal}}$, which is then combined with the dark diode via ideal superposition. As a result, the short-circuit current is essentially imposed by construction. The computed figures of merit are
  $$J_{sc}=38.1240\;\mathrm{mA}/{\mathrm{cm}}^{2},\;V_{oc}=652.405\;\mathrm{mV},\;\mathrm{FF}=0.83841.$$
- PNN ($p^{+}$–*n*–$n^{+}$ TM model): The same g(x) is included throughout the quasi-neutral regions through the transfer relations, so that recombination and boundary/interface losses compete with distributed generation during transport. In this case, the collected current emerges as a model output. The resulting photovoltaic parameters are
  $$J_{sc}=37.8595\;\mathrm{mA}/{\mathrm{cm}}^{2},\;V_{oc}=651.074\;\mathrm{mV},\;\mathrm{FF}=0.83817.$$

The difference in $J_{sc}$ between PN and PNN is consistent with the modeling assumptions of each approach. In the PN reduction, once $J_{\mathrm{ph}}^{\mathrm{ideal}}$ is specified, carrier collection is assumed to be perfect, and optical losses enter only through the ideal optical current defined in Equation (8). In contrast, the PNN formulation treats g(x) as a distributed source within the transport problem itself; recombination in the emitter, base, and rear-field regions, together with boundary losses, directly reduces the collected current. The PNN value corresponds to a collection ratio of 0.9931, which is approximately 99.31% of the ideal optical current. The small changes in $V_{oc}$ and FF show that the main difference in this benchmark is the treatment of carrier collection.

This benchmark confirms that the TM framework can use tabulated internal sources while preserving numerical stability. It also separates imposed collection in the PN reduction from predicted collection in the PNN model. This distinction is important before the same external series and shunt losses are applied to both transport descriptions. These controlled benchmarks are numerical verification and internal-consistency tests of the transfer-matrix implementation; they are not intended to constitute an independent experimental validation of the model.

### 4.3. Effect of $R_{s}$ and $R_{sh}$ Under Tabulated g(x)

This subsection assesses how lumped resistive losses modify the illuminated *J*–*V* response when the optical input is prescribed as a tabulated generation profile g=g(x). The purpose is not to fit experimental data yet but rather to verify that both reduced models (PN and PNN) remain circuit-consistent once the same external electrical losses are applied.

Both models use the same tabulated profile g(x), but they incorporate it in fundamentally different ways.

(i) PN (textbook $p^{+}/n$ superposition). The classical $p^{+}/n$ model uses the ideal photogenerated current density defined in Equation (8) and combines it with the dark diode through ideal superposition,

(35) $$J_{\mathrm{core},\mathrm{PN}}\left(V_{d}\right)=J_{\mathrm{ph}}^{\mathrm{ideal}}-J_{0,\mathrm{pn}}\;\left(\exp\;\left(\frac{V_{d}}{V_{T}}\right)-1\right),$$

where $J_{0,\mathrm{pn}}$ is the PN dark saturation-current density and $V_{T}$ is the thermal voltage. In this representation, once $J_{\mathrm{ph}}^{\mathrm{ideal}}$ is fixed, the short-circuit current is essentially imposed ($J_{sc}\approx J_{\mathrm{ph}}^{\mathrm{ideal}}$), and optical effects enter only through the scalar optical current in Equation (8).

(ii) PNN (transfer-matrix propagation with internal sources). In the $p^{+}$–*n*–$n^{+}$ TM model, illumination is not reduced to a single number. Instead, the source is propagated across the quasi-neutral regions through the layer transfer relations (Equation (5)), so that recombination and boundary/interface losses compete with the distributed generation during transport. Consequently, the same g(x) may produce a smaller collected current than q∫gdx; i.e., $J_{sc}$ becomes a model output rather than an imposed quantity.

To compare both transport cores under the same external electrical losses, Equation (18) is applied without modifying either diffusion–reaction model. The intrinsic PN and PNN current densities, denoted by $J_{\mathrm{core},\mathrm{PN}}$ and $J_{\mathrm{core},\mathrm{PNN}}$, are first calculated on the same grid of internal diode voltages $V_{d}$. For each core, Equation (18) then gives the terminal current density $J_{\mathrm{term}}$ and the terminal voltage $V_{\mathrm{term}}$. The resulting pairs $\left(V_{\mathrm{term}},J_{\mathrm{term}}\right)$ define the external current–voltage curve. The numerical procedure is summarized in Section 3.3.

**Figure 4:**
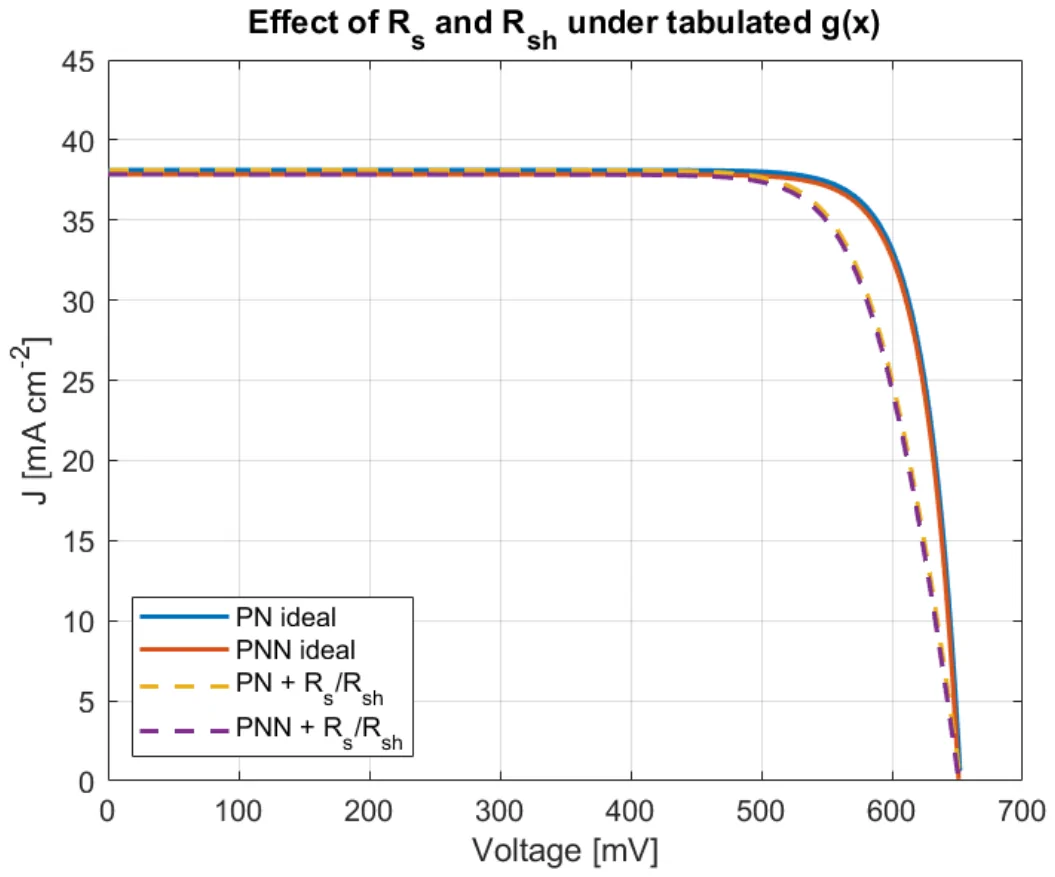
Forward benchmarks under generation profiles g(x): comparison of PN and PNN *J*–*V* curves for ideal and resistive cases.

The intrinsic PN and PNN curves, including their photovoltaic metrics, are the same as those reported in Section 4.2. They are included in Figure 4 only as reference curves before applying the external electrical losses.

Introducing representative lumped losses, $R_{s}=1\;\Omega \;\cdot \;{\mathrm{cm}}^{2}$ and $R_{sh}=2\times 10^{4}\;\Omega \;\cdot \;{\mathrm{cm}}^{2}$, both models display the expected degradation of the fill factor while leaving $J_{sc}$ and $V_{oc}$ nearly unchanged:

$$\mathrm{PN}:\;J_{sc}=38.1221\;\mathrm{mA}/{\mathrm{cm}}^{2},\;V_{oc}=652.383\;\mathrm{mV},\;\mathrm{FF}=0.78469,$$

$$\mathrm{PNN}:\;J_{sc}=37.8576\;\mathrm{mA}/{\mathrm{cm}}^{2},\;V_{oc}=651.053\;\mathrm{mV},\;\mathrm{FF}=0.78471.$$

This benchmark confirms that (i) the difference between the PN and PNN illumination treatments appears mainly in $J_{sc}$ because carrier collection is imposed in PN and calculated in PNN; and (ii) the same parametric series–shunt embedding can be applied to both intrinsic responses, allowing a consistent comparison and subsequent calibration.

### 4.4. Calibration of the Experimental 180 µm Reference Cell

Before inverse design, the PNN model was calibrated against the reported photovoltaic figures of merit of the 180 µm experimental reference cell. The fitted parameters were $C_{g}=1.0675$, $S_{\mathrm{front}}=S_{\mathrm{rear}}=S_{\mathrm{eff}}=1.469\times 10^{4}\;\mathrm{cm}\;s^{-1}$, and $R_{s,\mathrm{other}}=0.2984\;\Omega \;{\mathrm{cm}}^{2}$, while $\tau_{\mathrm{SRH}}=1.5\times 10^{-3}\;s$ and $R_{\mathrm{sh}}=2.0\times 10^{4}\;\Omega \;{\mathrm{cm}}^{2}$ were retained. Including the sheet and contact contributions gives $R_{s,\mathrm{total}}=1.008\;\Omega \;{\mathrm{cm}}^{2}$.

The calibrated model reproduces $J_{sc}=39.20\;\mathrm{mA}/{\mathrm{cm}}^{2}$, $V_{oc}=653.1\;\mathrm{mV}$, FF=0.783, and η=20.05%. No calibration parameter was modified during the subsequent optimization. The fitted $C_{g}$ should therefore be interpreted as an effective broadband generation-scale parameter of the reduced model rather than as a wavelength-independent physical correction to the measured optical response.

**Figure 5:**
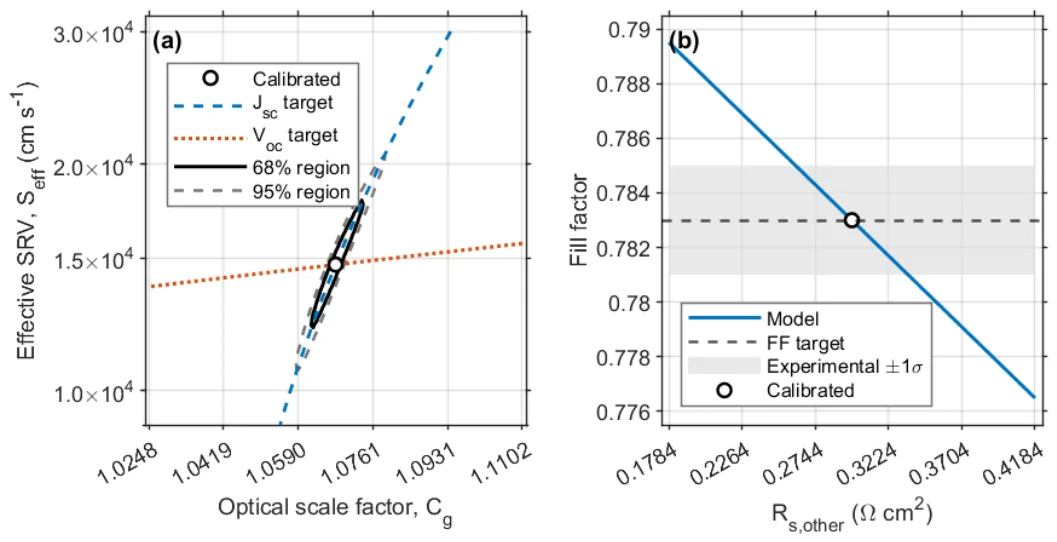
Calibration-sensitivity analysis of the experimental n-PERT reference cell. (**a**) Sensitivity of the first calibration stage to the optical scale factor $C_{g}$ and the common effective surface-recombination velocity $S_{\mathrm{eff}}$. The $J_{sc}$ and $V_{oc}$ curves indicate the reported experimental targets, and the 68% and 95% contours indicate the nominal joint regions obtained from the reported experimental uncertainties. (**b**) Sensitivity of the fill factor to the non-geometrical series-resistance contribution $R_{s,\mathrm{other}}$. The shaded region represents the reported experimental uncertainty in the fill factor. The markers indicate the calibrated solutions.

The sensitivity analysis was then used to determine how strongly the fitted parameters were constrained by the reported photovoltaic metrics. Figure 5 summarizes both calibration stages. For the first stage, the exact $J_{sc}=39.2$ mA/cm^2^ and $V_{oc}=653.1$ mV target contours intersected at $C_{g}=1.0675$ and $S_{\mathrm{eff}}=1.469\times 10^{4}$ cm/s, reproducing the calibrated solution.

The joint uncertainty region was elongated, however, showing that the two parameters remain strongly correlated. A local weighted sensitivity analysis performed using $C_{g}$ and $\log_{10}\left(S_{\mathrm{eff}}\right)$ gave a correlation coefficient of approximately 0.982. Under the uncertainty interpretation defined in Section 2.6, the nominal 95% joint region projected approximately onto $1.058\leq C_{g}\leq 1.079$ and $1.06\times 10^{4}\leq S_{\mathrm{eff}}\leq 2.06\times 10^{4}$ cm/s. These ranges are projections of a correlated joint region and should not be interpreted as independent parameter intervals.

For the second calibration stage, the fill factor decreased monotonically with increasing $R_{s,\mathrm{other}}$. The reported range FF=0.783±0.002 corresponded approximately to $0.262\leq R_{s,\mathrm{other}}\leq 0.335\;\Omega \;{\mathrm{cm}}^{2}$ when $C_{g}$ and $S_{\mathrm{eff}}$ were kept fixed. The calibrated value, $R_{s,\mathrm{other}}=0.2984\;\Omega \;{\mathrm{cm}}^{2}$, lies within this range.

### 4.5. Optimization of the Calibrated 180 µm Reference Geometry

The first inverse-design application keeps the experimentally calibrated wafer thickness fixed at 180 µm and optimizes only the front surface concentration, base doping, and rear surface concentration. This comparison isolates the effect of the diffusion-related design variables while preserving the geometry and loss calibration of the reference cell.

The calibrated reference gives $J_{sc}=39.20\;\mathrm{mA}/{\mathrm{cm}}^{2}$, $V_{oc}=653.1\;\mathrm{mV}$, a fill factor of 0.783, and an efficiency of 20.05%. After optimization, the efficiency increases to 20.63%, corresponding to an absolute gain of 0.58 percentage points. The optimized solution has a lower extracted short-circuit current density, $J_{sc}=38.54\;\mathrm{mA}/{\mathrm{cm}}^{2}$, but this reduction is compensated by an increase in $V_{oc}$ to 661.5 mV and in the fill factor to 0.809. This trade-off produces a higher maximum output power. The calibrated and optimized electrical performance is summarized in Table 2.

**Figure 6:**
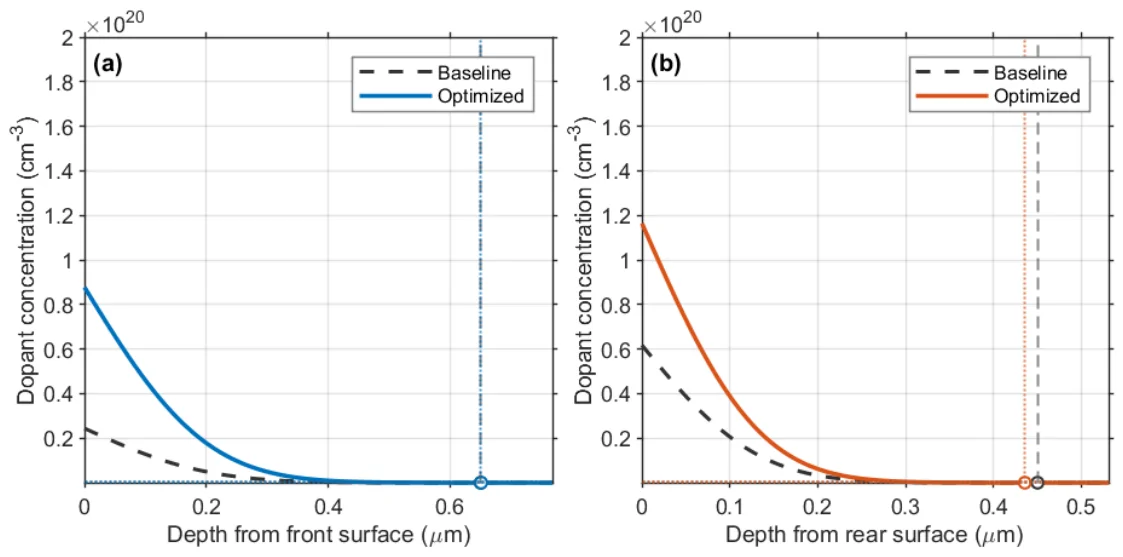
Baseline and optimized dopant profiles for the fixed 180 µm reference geometry. (**a**) Front $p^{+}$ emitter profile, with $W_{E}=0.650\;\mu m$ for the baseline design and $W_{E}=0.651\;\mu m$ for the optimized design. (**b**) Rear $n^{+}$ BSF profile, with $W_{\mathrm{BSF}}=0.450\;\mu m$ for the baseline design and $W_{\mathrm{BSF}}=0.436\;\mu m$ for the optimized design.

Figure 6 shows how the optimized design modifies the physically constrained diffusion profiles. The front and rear surface concentrations increase relative to the calibrated reference together with the base concentration. Because the junction depths are derived from the complementary-error-function profiles rather than optimized independently, these concentration changes produce only small changes in $W_{E}$ and $W_{\mathrm{BSF}}$. Once the reduced model is anchored to the experimental reference point, the optimization identifies a different balance between carrier collection, recombination, and resistive losses. In particular, the optimizer accepts a moderate reduction in $J_{sc}$ in exchange for higher $V_{oc}$ and fill factor.

For the 180 µm design, the total series resistance decreases from 1.008 to 0.570 Ω cm^2^. This reduction is almost entirely associated with the sheet-resistance contribution, which decreases from 0.708 to 0.271 Ω cm^2^. The contact-resistance contribution is much smaller, decreasing from approximately $1.13\times 10^{-3}$ to $1.99\times 10^{-4}$ Ω cm^2^, while $R_{s,\mathrm{other}}=0.2984\;\Omega$ cm^2^ remains fixed.

The quantitative loss decomposition shows that the optimization gain is not produced by the resistive improvement alone. For the 180 µm case, the remaining carrier-transport and collection changes contribute approximately +1.261 percentage points to the efficiency change, while the reduction in sheet-resistance losses contributes +0.639 percentage points. The contact-resistance contribution is negligible (+0.001 percentage points). These positive contributions are partially offset by increased penalties associated with Auger recombination (−0.397 percentage points), band-gap narrowing (−0.653 percentage points), and the fixed surface-recombination boundaries (−0.271 percentage points). Their combined balance gives the net efficiency increase of approximately +0.581 percentage points.

### 4.6. Robustness of the Optimization Framework for Prescribed Wafer Thicknesses

To evaluate the robustness of the inverse-design procedure under different geometrical constraints, independent optimizations were performed for prescribed wafer thicknesses of 160, 180, and 200 µm. The wafer thickness was not included in the design vector. For each case, the front surface concentration, base doping, and rear surface concentration were re-optimized using the same transport model, parameter bounds, genetic-algorithm configuration, and local-refinement budget.

The optimization increases the conversion efficiency for all three geometries. The absolute gains are approximately 0.56–0.60 percentage points for 160, 180, and 200 µm, respectively. In every case, the optimized solution shows a moderate reduction in extracted $J_{sc}$, between approximately 0.64 and 0.68 mA/cm^2^ together with an increase of approximately 7.9–8.9 mV in $V_{oc}$ and approximately 0.026 in the fill factor. The efficiency improvement is therefore not caused by higher optical generation but rather by a more favorable electrical trade-off in the terminal current–voltage response. The baseline and optimized electrical performance for the three prescribed wafer thicknesses is summarized in Table 3.

The corresponding optimized design variables and derived quantities are summarized in Table 4.

**Figure 7:**
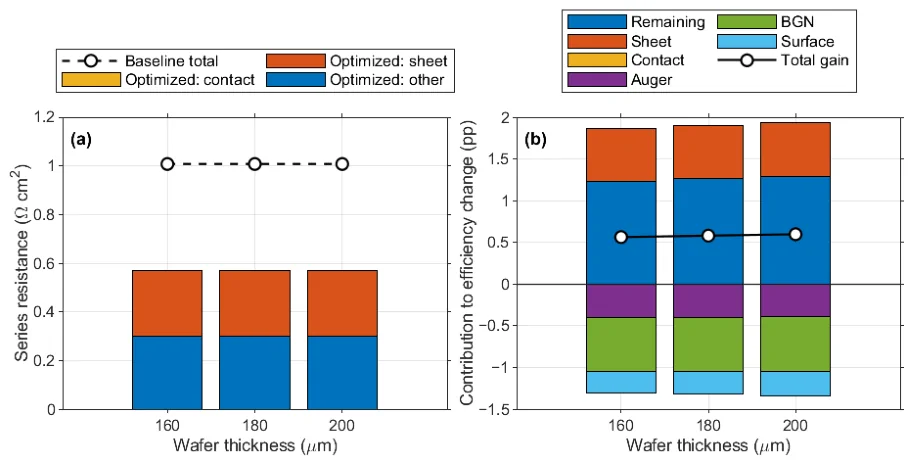
Quantitative loss decomposition for the prescribed wafer-thickness scenarios. (**a**) Series-resistance decomposition of the optimized designs into the fixed non-geometrical contribution, sheet resistance, and contact resistance; the dashed black line with open circles indicates the total series resistance of the corresponding baseline designs. (**b**) Interaction-aware decomposition of the calculated efficiency change into transport/collection, sheet-resistance, contact-resistance, Auger-recombination, band-gap-narrowing, and surface-recombination contributions. In panel (**b**), the black markers indicate the net efficiency gain obtained from the complete model.

The optimized designs form a smooth and physically related family. Figure 7 shows that this regularity also extends to the underlying loss balance. In all three thickness scenarios, optimization substantially reduces the sheet-resistance contribution, whereas the contact contribution remains very small. The interaction-aware efficiency decomposition also shows the same qualitative competition among improved transport/collection and sheet resistance and increased Auger, bandgap-narrowing, and surface-recombination penalties. The similarity of this balance across the three prescribed thicknesses supports the physical consistency of the optimized solution family.

Within the investigated interval, the baseline and optimized efficiencies both increase with wafer thickness, which is mainly because of the higher extracted current available in the thicker absorbers. The monotonic thickness trend reflects the present single-pass front-illumination model, which uses the measured wavelength-dependent front reflectance but does not explicitly resolve surface texture, light trapping, rear reflection, or bifacial rear illumination. The prescribed-thickness comparison therefore serves as a robustness test of the optimization framework. A fabrication-oriented thickness optimization would require a more complete optical description and wavelength-resolved experimental assessment [46,47].

## 5. Discussion

The transfer-matrix formulation combines carrier transport, distributed optical generation, electrical losses, experimental calibration, and numerical optimization within one reduced model. The limiting cases verify the treatment of boundary recombination and the explicit rear-field region, while the tabulated-generation benchmark shows that carrier collection can be predicted from an internal source instead of being imposed through $J_{sc}$. Because the layer matrices and source vectors are calculated before the voltage sweep, the forward model remains suitable for repeated evaluations in calibration and inverse design.

Calibration links the reduced model to the measured n-PERT device before the diffusion profiles are optimized. The fitted optical scale, effective surface recombination, and non-geometrical series resistance represent the combined response at the resolution of the one-dimensional model. After calibration, the optimization balances competing transport, recombination, and resistive mechanisms: the higher-doped profiles reduce sheet resistance but increase the penalties associated with Auger recombination and band-gap narrowing [48,49]. The interaction-aware loss decomposition shows that the efficiency improvement results from this coupled balance rather than from a single dominant mechanism. The complementary-error-function profiles provide an additional physical constraint by linking surface concentration and junction depth through the diffusion parameters.

The fitted $C_{g}$ and $S_{\mathrm{eff}}$ are effective parameters of the reference cell. The sensitivity analysis shows that they are locally constrained but strongly correlated, so $J_{sc}$ and $V_{oc}$ do not establish global parameter uniqueness. Application of the framework to another device architecture would therefore require device-specific recalibration.

The independent 160, 180, and 200 µm optimizations produce a smooth family of solutions. The emitter and rear-field depths change only slightly, the optimized doping concentrations vary gradually, and the total series resistance remains almost constant. These trends support the numerical robustness of the inverse-design procedure under different prescribed geometries. The present front-side optical model does not explicitly describe texture, light trapping, rear reflection, or rear illumination [50]; therefore, the thickness scenarios are used to test the transport and optimization framework under controlled geometrical changes.

As an additional wavelength-resolved diagnostic, the single-pass optical–transport response was compared with the previously reported front-side EQE of the same reference cell [24]. The EQE data were not used for parameter fitting. Because $C_{g}$ represents an effective broadband generation correction, the spectral diagnostic was evaluated with $C_{g}=1$ while retaining the calibrated semiconductor transport and recombination parameters. The RMSE is 5.15 percentage points over 300–900 nm and 19.21 percentage points over 300–1200 nm. The larger long-wavelength discrepancy reflects the absence of light trapping, rear reflection, and texture-enhanced optical path lengths in the present single-pass description.

The framework provides an intermediate modeling level between analytical solar-cell models and multidimensional TCAD. Its main uses are rapid physics-based screening, experimental calibration, and an interpretation of parameter trends. Future extensions could obtain the required R(λ) directly from an independent optical solver, for example a multilayer optical transfer-matrix model for sufficiently characterized planar stacks, or from ray-tracing or hybrid optical treatments for textured silicon devices. Such extensions could then be coupled to the present carrier-transport framework together with distributed grid resistance and two-dimensional current collection [51,52]. Additional spectral-response, temperature-dependent, or lifetime measurements would further improve parameter identification [53,54,55].

## 6. Conclusions

A physics-based transfer-matrix framework was developed for carrier transport, calibration, and optimization in layered bifacial $p^{+}$–*n*–$n^{+}$ crystalline-silicon solar cells. The formulation propagates normalized minority-carrier concentration and diffusion flux through explicit emitter, base, and rear-field transport regions and incorporates spatially varying optical generation through regional source vectors. Surface recombination, doping-dependent transport, Auger recombination, band-gap narrowing, sheet and contact resistances, and external circuit losses are treated within the same workflow.

The model was verified in limiting transport cases and with tabulated generation profiles before being calibrated against an experimental bifacial n-PERT reference cell. Physics-constrained optimization at prescribed wafer thicknesses of 160, 180, and 200 µm produced a smooth family of solutions and revealed a consistent physical trade-off in the diffused crystalline-silicon regions. The optimized designs favor higher front, base, and rear dopant concentrations while the physically derived emitter and rear-field junction depths remain nearly unchanged. For the 180 µm case, the main resistive benefit is the reduction in sheet resistance, whereas the contact-resistance contribution is negligible. This benefit, together with improved carrier transport and collection, is partially offset by stronger Auger-recombination, band-gap-narrowing, and surface-recombination penalties. The resulting efficiency improvement therefore arises from a quantitatively resolved balance between conductivity and recombination rather than from maximizing doping or carrier collection independently.

Building on the established transfer-matrix method, the methodological contribution of this paper is the integration of explicit emitter/base/rear-field carrier propagation, distributed optical generation, surface-recombination boundary conditions, physics-linked diffusion profiles, doping-dependent transport and electrical losses, experimental calibration, and global optimization into a single reduced-order inverse-design workflow.

The optimized quantities are specific to the experimentally anchored reference cell. The broader value of the framework lies in exposing the coupled physical trends that arise during inverse design while retaining a compact layer-wise mathematical description. Future work should couple the transport model with more complete optical and distributed-metallization descriptions, extend the calibration to high-efficiency reference devices with additional spectral and recombination data, and quantitatively benchmark the computational performance of the reduced-order formulation against established solar-cell simulation tools.

## Figures and Tables

**Table 1:** Forward benchmark summary for constant generation $g\left(x\right)=g_{0}$. Values reported as $\left(J_{sc}\;\left[\mathrm{mA}/{\mathrm{cm}}^{2}\right],\;V_{oc}\;\left[\mathrm{mV}\right],\;\mathrm{FF}\right)$.

Case	PN			PNN		
	$J_{\mathrm{sc}}$	$V_{\mathrm{oc}}$	**FF**	$J_{\mathrm{sc}}$	$V_{\mathrm{oc}}$	**FF**
A: baseline	39.2000	653.116	0.83854	37.7553	650.092	0.83801
B: $S_{n},S_{p}\to \infty$	39.2000	594.358	0.82704	35.8049	614.133	0.83112
C: $S_{n},S_{p}=10^{6}$ cm/s	39.2000	594.374	0.82705	35.8652	614.977	0.83129
D: thin base ($W_{B}=60\;µm$)	39.2000	653.116	0.83854	13.2396	647.731	0.83754

**Table 2:** Calibrated reference and optimized electrical performance for the fixed 180 µm wafer geometry.

Quantity	Calibrated Reference	Optimized Design
$J_{sc}$ (mA/cm^2^)	39.20	38.54
$V_{oc}$ (mV)	653.1	661.5
Fill factor	0.783	0.809
$R_{s,\mathrm{total}}$ ($\Omega \;{\mathrm{cm}}^{2}$)	1.008	0.570
Efficiency (%)	20.05	20.63

**Table 3:** Baseline and optimized electrical performance for prescribed wafer thicknesses.

Wafer Thickness (µm)	Design	$J_{\mathrm{sc}}$ (mA/cm^2^)	$V_{\mathrm{oc}}$ (mV)	Fill Factor	Efficiency (%)
160	Baseline	38.96	653.4	0.783	19.94
160	Optimized	38.32	661.3	0.809	20.51
180	Baseline	39.20	653.1	0.783	20.05
180	Optimized	38.54	661.5	0.809	20.63
200	Baseline	39.40	652.8	0.783	20.13
200	Optimized	38.72	661.7	0.809	20.73

**Table 4:** Optimized design variables and derived quantities for prescribed wafer thicknesses.

Quantity	160 µm	180 µm	200 µm
$N_{A,s}$ (cm^−3^)	$8.73\times 10^{19}$	$8.78\times 10^{19}$	$8.81\times 10^{19}$
$N_{D,B}$ (cm^−3^)	$2.91\times 10^{15}$	$2.98\times 10^{15}$	$3.05\times 10^{15}$
$N_{D,s}^{\mathrm{rear}}$ (cm^−3^)	$1.16\times 10^{20}$	$1.16\times 10^{20}$	$1.17\times 10^{20}$
$W_{E}$ (µm)	0.651	0.651	0.650
$W_{B}$ (µm)	158.91	178.91	198.91
$W_{\mathrm{BSF}}$ (µm)	0.436	0.436	0.435
$R_{s,\mathrm{total}}$ ($\Omega \;{\mathrm{cm}}^{2}$)	0.571	0.570	0.569

## Data Availability

The data and MATLAB code supporting the findings of this paper are available in the Supplementary Materials. Additional information is available from the corresponding author upon reasonable request.

## Supplementary Materials

The following supporting information can be downloaded at: https://www.mdpi.com/article/10.3390/nano16181187/s1, Supplementary File S1 (ZIP): Supplementary Note S1, General Transfer-Matrix Derivation, Device Closure, and Source Quadrature; README file; MATLAB implementation of the transfer-matrix model, including the forward-calculation, experimental-calibration, and fixed-thickness-optimization scripts and the main solver; and the final depth-dependent optical-generation data used in the revised calculations.

## Appendix A. Model and Optimization Parameters

Table A1, Table A2 and Table A3 collect the principal physical, geometrical, contact, and numerical parameters used in the final modeling workflow. The constitutive relations for carrier mobility, band-gap narrowing, and Auger recombination are described in Section 2.5. In the optimization stage, $N_{A,s}$, $N_{D,B}$, and $N_{D,s}^{\mathrm{rear}}$ are design variables; $W_{E}$ and $W_{\mathrm{BSF}}$ are derived from the corresponding diffusion profiles; and $d_{0}$ is prescribed independently for each thickness scenario.

**Table A1:** Reference physical, geometrical, and electrical input parameters used for the calibrated n-PERT device.

Parameter	Symbol	Value
Operating temperature	*T*	298.15K
Effective conduction-band density	$N_{c}$	$2.790\times 10^{19}\;{\mathrm{cm}}^{-3}$
Effective valence-band density	$N_{v}$	$1.042\times 10^{19}\;{\mathrm{cm}}^{-3}$
Electron mobility	$\mu_{n}$	Arora model
Hole mobility	$\mu_{p}$	Arora model
Reference front surface concentration	$N_{A,s}^{\mathrm{ref}}$	$2.44\times 10^{19}\;{\mathrm{cm}}^{-3}$
Reference base concentration	$N_{D,B}^{\mathrm{ref}}$	$8.436\times 10^{14}\;{\mathrm{cm}}^{-3}$
Reference rear surface concentration	$N_{D,s}^{\mathrm{rear},\mathrm{ref}}$	$6.17\times 10^{19}\;{\mathrm{cm}}^{-3}$
Reference emitter depth	$W_{E}^{\mathrm{ref}}$	0.65 µm
Reference rear-field depth	$W_{\mathrm{BSF}}^{\mathrm{ref}}$	0.45 µm
Reference wafer thickness	$d_{0}^{\mathrm{ref}}$	180 µm
Front finger pitch and width	$s_{E},\;w_{E}$	1.5 mm, 40 µm
Rear finger pitch and width	$s_{R},\;w_{R}$	1.3 mm, 60 µm
Simulated area	*A*	$1\;{\mathrm{cm}}^{2}$
Incident power density	$P_{\mathrm{in}}$	$0.1\;W\;{\mathrm{cm}}^{-2}$
Auger coefficient for electrons	$C_{n}$	$2.80\times 10^{-31}\;{\mathrm{cm}}^{6}s^{-1}$
Auger coefficient for holes	$C_{p}$	$9.90\times 10^{-32}\;{\mathrm{cm}}^{6}s^{-1}$

**Table A2:** Electrical contact-model parameters used in the calibrated optimization workflow.

Parameter	Symbol	Front	Rear
Reference specific contact resistivity	$\rho_{c,E}^{\mathrm{ref}}$, $\rho_{c,R}^{\mathrm{ref}}$	$1.0\times 10^{-4}$	$1.0\times 10^{-4}$
Reference surface concentration	$N_{E}^{\mathrm{ref}}$, $N_{R}^{\mathrm{ref}}$	$1.0\times 10^{19}$	$1.0\times 10^{19}$
Doping exponent	$m_{E}$, $m_{R}$	1.5	1.5
Lower contact-resistivity limit	$\rho_{c,\min}$	$1.0\times 10^{-6}$	$1.0\times 10^{-6}$
Upper contact-resistivity limit	$\rho_{c,\max}$	$1.0\times 10^{-1}$	$1.0\times 10^{-1}$

Specific contact-resistivity values are expressed in Ω cm^2^, and reference surface concentrations in cm^−3^.

**Table A3:** Design-variable bounds and hybrid optimization settings. Dopant concentrations are represented internally using base-10 logarithms.

Item	Symbol or Method	Value
Front surface concentration	$N_{A,s}$	$10^{17}$–$5\times 10^{20}$ cm^−3^
Base concentration	$N_{D,B}$	$10^{14}$–$10^{17}$ cm^−3^
Rear surface concentration	$N_{D,s}^{\mathrm{rear}}$	$10^{18}$–$5\times 10^{20}$ cm^−3^
Prescribed wafer thicknesses	$d_{0}$	160, 180, and 200 µm
Emitter-depth bounds	$W_{E}$	0.05–4.0 µm
Rear-field-depth bounds	$W_{\mathrm{BSF}}$	0.02–3.0 µm
Minimum base thickness	$W_{B}$	5.0 µm
Global algorithm	MATLAB ga	Genetic algorithm
Population size	—	80
Maximum generations	—	100
Maximum stall generations	—	30
Function tolerance	—	$10^{-6}$
Initial GA design	$\theta_{\mathrm{ref}}$	Calibrated reference included
Random-number generator	MATLAB rng	rng(1,’twister’)
Parallel fitness evaluation	MATLAB UseParallel	Enabled
Local refinement	MATLAB fmincon	SQP
Maximum local function evaluations	—	500
Optimality tolerance	—	$10^{-8}$
Step tolerance	—	$10^{-10}$
