# Supplementary material for: Physics-Constrained Transfer-Matrix Optimization of Diffused Regions in Bifacial p+–n–n+ Crystalline-Silicon Solar Cells
Source: Nanomaterials (Basel). 2026 Sep 20;16(18):1187. doi: 10.3390/nano16181187 (PMC13610816; doi:10.3390/nano16181187)
Supplement: Supplementary file 1 [file nanomaterials-16-01187-s001.zip › Supplementary material.pdf]

Supplementary Materials

Physics-Constrained Transfer-Matrix Optimization of Diffused Regions in Bifacial p^+–n–n^+ Crystalline-Silicon Solar Cells

Pablo Ferrada[1,2,*] and Carlos Portillo[3]

[1] Departamento de Ingeniería, Universidad Loyola Andalucía, Av. de las Universidades 2, Dos Hermanas, 41704 Sevilla, Spain

[2] Instituto de Investigación en Energía, Tecnología y Sostenibilidad (LETS), Universidad Loyola Andalucía, Sevilla, Spain

[3] Centro de Desarrollo Energético Antofagasta (CDEA), Universidad de Antofagasta, Av. Angamos 601, 1270300 Antofagasta, Chile

[*] Correspondence: pdferrada@uloyola.es

Supplementary Note S1. General Transfer-Matrix Derivation, Device Closure, and Source Quadrature

This supplementary note presents the formal derivation of the operational layer relation used in Section 2.1 of the main manuscript. It also gives the extension to an arbitrary number of transport regions, the explicit emitter–base–BSF closure, and the numerical quadrature used for tabulated generation profiles.

Starting from Equation (1) of the main manuscript and using the definitions of u_r and L_r in Equation (2) of the main manuscript, the normalized diffusion–reaction equation in region r is

$$\frac{d^2 u_r}{dx'^2} = \frac{u_r}{L_r^2} - \frac{g_r(x')}{D_r c_{0,r}}. \tag{S1}$$

Here, x' is the local depth measured from the start of region r. Using the state vector defined in Equation (3) of the main manuscript, Equation (S1) can be written as the first-order system

$$\frac{d}{dx'}\mathbf{s}_r(x') = A_r \mathbf{s}_r(x') + \mathbf{f}_r(x'), \qquad A_r = \begin{pmatrix} 0 & \dfrac{1}{D_r c_{0,r}} \\ \dfrac{D_r c_{0,r}}{L_r^2} & 0 \end{pmatrix}, \qquad \mathbf{f}_r(x') = \begin{pmatrix} 0 \\ -g_r(x') \end{pmatrix}. \tag{S2}$$

The matrix A_r contains the constant transport coefficients of region r, and the column vector $\mathbf{f}_r$ contains the local optical source. At a local boundary position $x' = x'_\Gamma$, a general linear condition can be written as

$$\alpha_\Gamma u_r(x'_\Gamma) + \beta_\Gamma G_r(x'_\Gamma) = \delta_\Gamma, \tag{S3}$$

where α_Γ, β_Γ, and δ_Γ are prescribed coefficients. This form includes fixed-concentration, fixed-flux, and Robin surface-recombination conditions.

When $g_r(x') = 0$, Equation (S1) becomes

$$\frac{d^2 u_r}{dx'^2} - \frac{u_r}{L_r^2} = 0, \tag{S4}$$

and its homogeneous solution is

$$u_r(x') = C_{1,r} \cosh\left(\frac{x'}{L_r}\right) + C_{2,r} \sinh\left(\frac{x'}{L_r}\right), \tag{S5}$$

where $C_{1,r}$ and $C_{2,r}$ are integration constants. For a physical propagation distance Δx, the corresponding fundamental matrix is

$$\exp(A_r \Delta x) = M_r(\Delta x), \tag{S6}$$

where $M_r(\Delta x)$ is defined in Equation (4) of the main manuscript. Applying the variation-of-constants formula to Equation (S2) gives the regional relation in Equations (5) and (6) of the main manuscript.

For a structure containing N transport regions, let $M_k = M_k(\ell_k)$ denote the complete transfer matrix of region k, and let $\mathbf{b}_k$ denote its source vector. Repeated substitution gives the global input–output relation

$$\mathbf{s}_{\text{out}} = M_{\text{tot}} \mathbf{s}_{\text{in}} + \mathbf{b}_{\text{tot}}, \tag{S7}$$

where $\mathbf{s}_{\text{in}}$ and $\mathbf{s}_{\text{out}}$ are the states at the left and right boundaries of the complete layered structure, respectively. The total transfer matrix and total source vector are

$$\begin{aligned} M_{\text{tot}} &= M_N M_{N-1} \cdots M_1, \\ \mathbf{b}_{\text{tot}} &= M_N M_{N-1} \cdots M_2 \mathbf{b}_1 \\ &\quad + M_N M_{N-1} \cdots M_3 \mathbf{b}_2 \\ &\quad + \cdots + M_N \mathbf{b}_{N-1} + \mathbf{b}_N. \end{aligned} \tag{S8}$$

Thus, M_{tot} propagates the input state through all regions, while $\mathbf{b}_{\text{tot}}$ adds the source contribution of each region after propagation through the regions located to its right.

For the p^+–n–n^+ device considered in this work, the complete regional matrices are written as $M_E = M_E(W_E)$, $M_B = M_B(W_B)$, and $M_S = M_S(W_{\text{BSF}})$. The subscripts E, B, and S denote the emitter, base, and BSF transport regions, respectively. For the emitter, the matrix and source vector are written as

$$M_E = \begin{pmatrix} e_{11} & e_{12} \\ e_{21} & e_{22} \end{pmatrix}, \qquad \mathbf{b}_E = \begin{pmatrix} b_{1E} \\ b_{2E} \end{pmatrix}, \tag{S9}$$

where e_{ij} are the four elements of the complete emitter matrix, and b_{1E} and b_{2E} are the two components of the emitter source vector.

The base and BSF regions are first constructed separately and then combined into a block extending from the base-side depletion edge to the rear surface:

$$M_{SB} = M_S M_B = \begin{pmatrix} m_{11} & m_{12} \\ m_{21} & m_{22} \end{pmatrix}, \qquad \mathbf{b}_{SB} = M_S \mathbf{b}_B + \mathbf{b}_S = \begin{pmatrix} b_{1SB} \\ b_{2SB} \end{pmatrix}. \tag{S10}$$

At the front surface, the boundary condition is $G_E(0) = \gamma_F u_E(0)$, where $\gamma_F = S_n c_{0,E}$ and S_n is the effective front surface-recombination velocity. At the emitter-side depletion edge, $u_E(W_E; V_d) = u_0(V_d)$, where V_d is the internal diode voltage and $u_0(V_d)$ is the normalized injection. Applying Equation (5) of the main manuscript gives

$$u_E(0; V_d) = \frac{u_0(V_d) - b_{1E}}{e_{11} + \gamma_F e_{12}}, \tag{S11}$$

$$G_E(W_E; V_d) = (e_{21} + \gamma_F e_{22})\, u_E(0; V_d) + b_{2E}. \tag{S12}$$

For the base–BSF block, the state at the base-side depletion edge is

$$\mathbf{s}_B(0; V_d) = \begin{pmatrix} u_0(V_d) \\ G_B(0; V_d) \end{pmatrix}.$$

At the rear surface, the boundary condition is $G_S(W_{\mathrm{BSF}}) = -\gamma_R u_S(W_{\mathrm{BSF}})$, where $\gamma_R = S_p c_{0,S}$ and S_p is the effective rear surface-recombination velocity. Substitution into Equation (S10) gives

$$G_B(0; V_d) = -\frac{(m_{21} + \gamma_R m_{11})\, u_0(V_d) + b_{2SB} + \gamma_R b_{1SB}}{m_{22} + \gamma_R m_{12}}. \tag{S13}$$

Although the device current is evaluated from the two fluxes at the main p^+/n depletion edges, the BSF contribution is included in $G_B(0; V_d)$. This flux is calculated with the combined matrix $M_{SB} = M_S M_B$, the combined source vector $\mathbf{b}_{SB}$, and the rear-surface boundary condition. Therefore, no separate BSF current term is required.

With nonzero source vectors, Equations (S12) and (S13) provide the illuminated depletion-edge fluxes used to calculate $J_{\mathrm{core}}(V_d; g)$. When the optical generation is set to zero, the same equations provide the dark fluxes used in Equation (15) of the main manuscript to calculate $J_d(V_d)$.

For tabulated optical generation, the source vector is evaluated with the trapezoidal rule. Let N_r be the number of local grid points in region r, and let $x'_j \in [0, \ell_r]$ be the local coordinate of grid point j. The generation value at this point is $g_{r,j} = g_r(x'_j) = g(a_r + x'_j)$, where a_r is the global

starting position of region r, as defined in Section 2.1 of the main manuscript. The contribution generated at x'_j must propagate through the remaining physical distance $\ell_r - x'_j$. Its propagated source contribution at the right boundary is

$$\mathbf{v}_{r,j} = M_r(\ell_r - x'_j) \begin{pmatrix} 0 \\ -g_{r,j} \end{pmatrix}, \tag{S14}$$

where $\mathbf{v}_{r,j}$ is a two-component column vector. The numerical source vector is then

$$\mathbf{b}_r \approx \sum_{j=1}^{N_r-1} \frac{x'_{j+1} - x'_j}{2} \left(\mathbf{v}_{r,j} + \mathbf{v}_{r,j+1}\right). \tag{S15}$$

The supplementary MATLAB implementation follows the same regional matrices, source vectors, boundary conditions, depletion-edge fluxes, and quadrature rule.
